# The global prevalence of autism spectrum disorder: A three-level meta-analysis

**DOI:** 10.3389/fpsyt.2023.1071181

**Published:** 2023-02-09

**Authors:** Oksana I. Talantseva, Raisa S. Romanova, Ekaterina M. Shurdova, Tatiana A. Dolgorukova, Polina S. Sologub, Olga S. Titova, Daria F. Kleeva, Elena L. Grigorenko

**Affiliations:** ^1^Center for Cognitive Sciences, Sirius University of Science and Technology, Sirius, Russia; ^2^Laboratory of Translational Developmental Sciences, Department of Psychology, Saint Petersburg State University, Saint Petersburg, Russia; ^3^Center for Bioelectric Interfaces, National Research University Higher School of Economics, Moscow, Russia; ^4^Department of Psychology, University of Houston, Houston, TX, United States; ^5^Department of Molecular and Human Genetics, Baylor College of Medicine, Houston, TX, United States; ^6^Child Study Center, Yale University, New Haven, CT, United States

**Keywords:** autism, prevalence, epidemiology, meta-analysis, systematic review

## Abstract

Autism spectrum disorder (ASD) is one the most disabling developmental disorders, imposing an extremely high economic burden. Obtaining as accurate prevalence estimates as possible is crucial to guide governments in planning policies for identification and intervention for individuals with ASD and their relatives. The precision of prevalence estimates can be heightened by summative analyses of the data collected around the world. To that end, we conducted a three-level mixed-effects meta-analysis. A systematic search of the Web of Science, PubMed, EMBASE, and PsycINFO databases from 2000 up to 13 July 2020 was performed, and reference lists of previous reviews and existing databases of prevalence studies were screened. Overall, 79 studies were included in the analysis of ASD and 59–in the analysis of previously existing relevant diagnoses: 30 for Autistic Disorder (AD), 15 for Asperger Syndrome (AS), and 14 for Atypical Autism (AA) and Pervasive Developmental Disorder - Not Otherwise Specified (PDD-NOS); these research reports covered the period from 1994 to 2019. Pooled prevalence estimates were 0.72% (95% CI = 0.61–0.85) for ASD, 0.25% (95% CI = 0.18–0.33) for AD, 0.13% (95% CI = 0.07–0.20) for AS, and 0.18% (95% CI = 0.10–0.28) for the combined group of AA and PDD-NOS. Estimates were higher (1) for the studies that used records-review surveillance rather than other designs; (2) in North America compared with other geographical regions; and (3) in high-income compared with lower-income countries. The highest prevalence estimates were registered in the USA. There was an increase in autism prevalence estimates over time. The prevalence was also significantly higher for children aged between 6 and 12 years compared to children under the age of 5 and over the age of 13 years.

**Systematic review registration:**

https://www.crd.york.ac.uk/prospero/display_record.php?ID=CRD42019131525, identifier CRD42019131525.

## 1. Introduction

Autism Spectrum Disorder (ASD) is a lifelong neurodevelopmental disorder characterized by difficulties in the social-communication domain, and the presence of restricted and/or repetitive interests and/or behavior (1, 2) manifested before the age of three. ASD has encompassed a number of diagnoses that, in previous versions of medical classifications, were clustered in the group of Pervasive Developmental Disorders (PDDs). However, by being a spectrum, this condition is also characterized by a high level of heterogeneity of the phenotypic manifestations, associated with wide variability in levels of intellectual and language development, intra-individual discrepancies in cognitive profiles (3, 4), and frequently reported comorbidity with other developmental disorders and psychiatric conditions (5). This combination of specific deficits and high comorbidity places ASD among the most disabling developmental disorders, imposing an extremely high economic burden. For example, in the USA, by 2025, the forecast annual direct and productivity costs of ASD could reach $461 billion (6).

Investigations into the prevalence of autism began as early as the 1960s–1970s (7–9), even before the inclusion of ASD in the international diagnostic classification and before the unification of ASD diagnostic criteria. ASD prevalence in these initial studies was estimated to be between 0.5 (9) and 0.7 (8) cases per 10,000 people. Since the 1970s, prevalence studies have been conducted across different regions, covering at least 37 countries worldwide, although the data are still lacking in many low- and middle-income countries (10). Notably, these studies have shown a dramatic increase in ASD prevalence estimates since the late 1900s/early 2000s. This time trend is particularly evident if we compare data obtained in cross-sectional monitoring programs that were developed in some counties to track changes in the prevalence of ASD. Thus, according to the Autism and Developmental Disabilities Monitoring (ADDM) Network launched by the Centers for Disease Control and Prevention (CDC) in the USA, the prevalence of ASD among 8-year-old children in 2000 was 67 per 10,000 (11), 90/10,000 in 2006 (12), and 145/10,000 in 2012 (13). The most recent report, dated 2018, revealed that ASD affects approximately 230 per 10,000 children (14), indicating that its prevalence has increased by 243% since the first ADDM Network study in 2000 (14). Previously published systematic reviews demonstrated that these discrepancies in estimates over time probably are not caused by an increase in true prevalence but are instead associated with changes and improvements in diagnostic categories, methodology, and quality of research, increased access to diagnostic and intervention services, increased awareness of ASD among both professional and non-professional communities, and acceptance of the fact that ASD can coexist with other forms of developmental disorders [for example, in (15–17)].

In addition to time trends, there are other sources of heterogeneity, such as geographical region, country income, implemented study design, diagnostic criteria used, age of the studied population, and other socio-demographic factors (18–20). The complex interaction between these factors makes obtaining a reliable, unified worldwide prevalence estimate challenging. However, obtaining pooled worldwide prevalence could play a critical role in indicating the public health burden of the disorder, allocating budgets for ASD in countries where prevalence studies have not been conducted or are just about to be performed, and detecting problems in the identification and diagnostics of ASD in countries where the data are well below the world mean estimates, and for revealing potential environmental risk factors for ASD in some regions.

The data from the most recent studies of global prevalence estimates are somewhat variable. For example, the systematic review by Zeidan et al. (18) showed that 71 studies and 99 prevalence estimates from 34 countries published from 2012 to 2021 report a median prevalence of 100 per 10,000 children; in the meta-analysis of Salari et al. (19) showed that 74 studies, published from 2008 to 2021 the pooled prevalence comprised 60 per 10,000.

Given the great heterogeneity encountered in the prevalence of ASD over time and inconsistency among previously published estimates, this study aimed to conduct a new meta-analysis. Therefore, the first aim of this research was to update the previous meta-analyses and systematic reviews. The second aim was to identify and analyze potential moderators regarding methodological aspects, geographical locations, age groups, and related socio-economical indexes that may influence the observed heterogeneity in prevalence estimates. Considering the multiple data that could be extracted from a single study, the present research also contributes to the current knowledge by applying a state-of-the-art, three-level meta-analytical approach to estimating the mean pooled prevalence of ASD and previously existing diagnoses of Autistic Disorder (AD), Asperger Syndrome (AS), Atypical Autism (AA), and Pervasive Developmental Disorder - Not Otherwise Specified (PDD-NOS), along with subgroup estimates for ASD.

## 2. Methods

### 2.1. Search strategy, selection, and inclusion criteria

This meta-analytical study is reported according to the Preferred Reporting Items for Systematic Review and Meta-Analyses statement [PRISMA; (21)]. The initial research protocol was registered at the International Prospective Register of Systematic Reviews (PROSPERO number: CRD42019131525). For searching relevant articles, we used the following bibliographic databases: Web of Science, PubMed, EMBASE, and PsycINFO and the following search terms: [autis* OR ASD OR (pervasive AND development* AND disorder) OR PDD OR Asperger] AND (prevalence OR incidence OR cross-sectional OR epidemiolog*), applying to headings and keywords for studies, published in English since 2000. This search algorithm was developed based on the analysis of the titles of 32 articles that had been previously manually identified. Before starting the systematic search, it was tested for its completeness to identify relevant citations. The initial search was performed on 9 March 2019. Due to the protracted work on the meta-analysis, we updated the database search on 13 July 2020 using the same search method, except that we narrowed the searches to 2019 onward. In order to avoid any missing studies, online repositories (22, 23) of ASD prevalence studies, the reference lists of relevant reviews, and studies eligible for full-text assessment were searched manually by the first author. After removing duplicates in Zotero, five reviewers (OIT, EMS, DK, OST, RR) screened titles and abstracts against inclusion criteria. For this stage, the database of citations was subdivided into five partly overlapping pools of articles. Thus, two reviewers assessed each article independently, and all discrepancies were discussed between all reviewers until a consensus was reached. Then seven reviewers (OIT, OST, DK, RR, EMS, TD, PS) assessed full-text articles on their eligibility for data extraction so that each article was analyzed by two reviewers independently. In the case of discrepancies, a final decision was accepted after discussion.

Studies were included if they met the following inclusion criteria: (a) a geographically and temporally defined population (description of study location and study years); (b) defined age of participants (or years of birth); (c) data about the prevalence of ASD or diagnostic subgroups (AD, AS, AA or PDD-NOS), (d) case identifications based on formal diagnostic procedures according to DSM or ICD criteria, or gold standard diagnostic tools, including different editions of the Autism Diagnostic Observation Schedule (ADOS) and Autism Diagnostic Interview (ADI).

We excluded studies that (a) were published in a language other than English; (b) not-full text articles (like letters and conference theses); (c) studies with no original data (i.e., different types of reviews and meta-analyses); (d) studies without information about the methods of case identification; (e) studies analyzing prevalence in not-general/special or high-risk populations.

Only those studies provided enough data for computing crude prevalence estimates with a sample size of more than 1,000 individuals, and point- or 1-year-period prevalence were included in the meta-analysis.

### 2.2. Data extraction

In the data extraction, most of the reviewers (OIT, EMS, RR, TD, PS) participated. As in the previous steps, to reduce the possibility of mistakes, data from each study was extracted and coded by two researchers independently. Discrepancies were resolved by consensus or by a first author. The following descriptive and methodological variables were coded from studies: first author, title, publication year, study location, country, geographical region, and income group as defined by the World Bank in (24) United Nation’s latest Human Development Index (25), study time, age of the at-risk population, study design, diagnostic group, diagnostic classification, screening and diagnostic tools, sample size, number of cases, prevalence rates (number per 10,000) with 95% confidence intervals (CIs), statistics by gender (sample size, number of cases, male-to-female ratio, prevalence estimates with 95% CIs) and percentage of cases with intellectual disability [Intelligence Quotient (IQ) ≤ 70]. In cases where non-crude prevalence estimates were extracted, we added additional values for the sample size or the number of cases for meta-analytical computations (depending on which value was weighted or adjusted in the study) so that prevalence would follow the simple formula: (the number of cases)/(population size)*10,000, to avoid inconsistencies between the original prevalence estimates from the studies and the estimates computed in the meta-analysis.

As for study time, when data were reported for a school year or in studies with multiphase population surveillance designs, the low band of time range was extracted. Study designs were classified depending on the source of data into the following categories: registers, administrative databases, health insurance databases, records-review surveillance, direct surveillance, and mixed designs (combining different methodologies).

As we hypothesized high heterogeneity among prevalence estimates for different countries, study years, and age groups if one article included multiple data for these specific categories, we considered it as containing multiple studies or data points, and more than one prevalence estimate was extracted.

### 2.3. Assessing risk of bias

Although in the study protocol, we originally intended to use the Joanna Briggs Institute critical appraisal tool for prevalence studies (26), in the process of working on this meta-analysis, we decided to change our decision in favor of assessing the risk of bias. For this aim, the Hoy Risk of Bias Tool [RoBT; (27)], designed specifically for prevalence studies, was chosen. The RoBT consists of 10 items, assessing both the external (items 1–4) and internal (items 5–10) validity of a study. Each item is scored on a binary scale: “0” means the absence of specific bias, “1” means its presence, so the scale has an overall maximum of 10, with higher scores reflecting a greater risk of bias. Summary scores of 0–3, 4–6, and 7–10 were assumed to indicate a “low,” “moderate,” and “high” risk of bias, respectively.

The risk of bias in the included studies was assessed by the same reviewers participating in the data extraction in a similar way: each study was assessed twice with the achievement of the consensus in the case of any conflict estimates.

### 2.4. Data analysis

To select the most optimal model for calculating the prevalence, we were guided by the following assumptions. First, because of anticipated heterogeneity influenced by the difference in study time, geographical location, diagnostic criteria, study design, and the age of the at-risk population, we selected the random-effect (compared with fixed-effect) model as better for included studies. Second, as we extracted multiple data points from one article contradicting the independence requirement (28), we assumed that an article could be seen as a higher-level variable in which multiple prevalence estimates could be nested. To deal with this intra-study dependency of prevalence estimates, we opted for the three-level model with random effects for data points obtained within one article to calculate pooled prevalence estimates (29, 30). To check that the three-level model has a better fit compared with the classical two-level random-effect model, we performed ANOVA. The final decision was based on the Akaike Information Criterion (AIC), Bayesian Information Criterion (BIC), and Log-likelihood absolute parameters with a preference for lower values along with the results of the likelihood ratio test. The Freeman–Tukey double-arcsine transformation was used to stabilize the variance of proportions before pooling the data with the multilevel random-effects model. Heterogeneity was assessed for each meta-analytical level using *I*^2^ with thresholds of ≥ 25%, ≥ 50%, and ≥ 75% indicating low, moderate, and high heterogeneity, respectively (31).

The effects of categorical variables (i.e., geographical region, country of origin, country income group, study time, age group of participants, study design, and risk of bias) were assessed using the multilevel random-effects moderator analysis of subgroups. In order to categorize study time for this analysis, we subdivided it into the following time periods: 1994–1999, 2000–2004, 2005–2009, 2010–2014, and 2015–2019. As for variables of the age group, the following levels were selected: 0–5, 6–12, and > 13 years. For examining continuous variables (i.e., age, HDI, study time), meta-regression analysis was implemented. In this case, age was put as the reported mean or weighted mean age or, if this was not provided, as the midpoint of the age range. Only those levels of variables with at least five data points were included in the subgroup and regression analysis.

As funnel plot asymmetry is not informative in the presence of very high heterogeneity, funnel plot asymmetry and Egger’s regression tests were not used to statistically examine publication biases (32); funnel plots were provided for illustration purposes only (Supplementary Figure 1).

All meta-analytical calculations were performed using the *rma.mv* function in *metafor* package (33) in R 4.2.1.

## 3. Results

### 3.1. Search results and sample characteristics of studies

**FIGURE 1:**
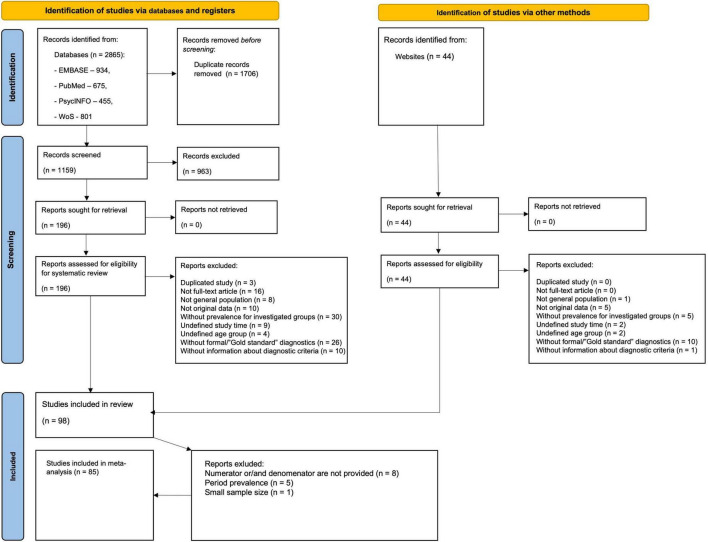
Search strategy. PRISMA flow diagram.

The flow diagram (Figure 1) depicts the literature search results and the study selection process. The initial search from databases identified 2,865 citations, of which 934 were from EMBASE, 675 from PubMed, 455 from PsycINFO, and 801 from WoS. There were 1,159 citations after the removal of duplicates. We retrieved 196 full-text articles assessed for eligibility after 963 citations were excluded after screening titles and abstracts. The additional search through online databases of ASD prevalence studies yielded 44 full-text articles, following manual searching among reference lists of systematic reviews and meta-analyses did not identify any additional citations. Altogether 98 articles were eligible for data extraction yielding 396 prevalence estimates. After data extraction, we additionally excluded 13 articles due to one of the following reasons: absence of denominator (population size) and/or numerator (number of ASD cases), period prevalence estimates, or small (< 1000) population size.

Overall, 85 articles with 317 extracted prevalence estimates were included in the meta-analysis, covering the period from 1994 to 2019 and 29 countries from Europe (139 estimates), Asia (98 estimates), North America (69 estimates), Oceania (6 estimates), South America (4 estimates), and Africa (1 estimate). No study from the Caribbean and Central America was included. More than half of the studies were completed in high-income countries (83.5%), mostly in the USA (23.5%). Most studies were based on administrative data (*N* = 20), while most prevalence estimates were obtained from registers (*n* = 121). In cross-sectional studies with direct surveillance, the most common screening tools were SCQ, ASSQ, and M-CHAT, while the most prevalent diagnostic instruments were: ADOS (including all editions), ADI-R, and CARS.

Thus, 220 estimates were included in meta-analyses of ASD, 57–of AD, 23–of AS. As for AA and PDD-NOS < 17 of the data points were identified (8 and 9, correspondingly), we have decided to merge them into one group. Hence, 271,518 (per 116,039,045) individuals were identified as having ASD, 4,820 (per 2,534,516)–AD, 553 (per 890,591)–AS, 953 (per 666,445)–AA/PDD-NOS. Among them (for whom data on intellectual functioning were available), the average percentage of individuals classified as having an intellectual disability (IQ ≤ 70) was 34.9 ± 13.5, 60.0 ± 23.8, 3.4 ± 4.5, 24.1 ± 21.6 for ASD, AD, AS, and AA/PDD, respectively. Across all studies with gender statistics, the mean male-to-female ratio was 4.3 (Med = 4.2, *SD* = 1.5) for ASD and 4.2 (Med = 4.2, *SD* = 1.6) for AD. For other subgroups, the ratio was more variable, and the gender ratio increased to 9.7 (Med = 6.2, *SD* = 8.0) for AS and 7.0 (Med = 4.3, *SD* = 3.0) for AA and PDD-NOS. There was clearly a wide variation in the prevalence estimates. For ASD, prevalence estimates ranged from 0.6 (34) to 313.3 per 10,000 (35) (Med = 47.7, *SD* = 58.8), for AD–from 0 (36) to 135.1 (37) (Med = 20.7, *SD* = 21.9), for AS–from 1.1 (38) to 51.9 (39) (Med = 7, *SD* = 12.4), for AA–from 0.5 (40) to 22.5 (41) (Med = 10.7, *SD* = 7.7), for PDD-NOS–from 4.3 (38) to 64.8 (42) (Med = 28.4, *SD* = 17.6) per 10,000.

### 3.2. Assessing risk of bias

The risk of biases for identified prevalence studies ranged from low to moderate, and only two studies were assessed as having high risk (*M* = 3.8, *SD* = 1.5). In 66 studies, target populations were not representative of the national population (item 1 of RoBT), which was the most commonly identified risk of bias. Also, many studies failed to demonstrate explicitly that the likelihood of non-response bias in at-risk populations was minimal and that the same mode of data collection was used for all subjects [items 4 (*N* = 62) and 8 (*N* = 58) of RoBT].

### 3.3. Model fit and global pooled prevalence

To check that the three-level models are better at capturing the variability of the data for analyzing diagnostic groups than classical two-level models, we performed ANOVA to compare the full models to models where level 3 was removed. Model-fit assessment (Supplementary Table 7) revealed that the data showed a significantly better fit to the full models compared to the reduced ones for all diagnostic groups, with the exception of the combined AA and PDD-NOS group. Despite the absence of statistically significant differences between full and reduced models for the AA and PDD-NOS group, we decided to proceed with a three-level model, as it accounts for multiple estimates from a single study.

**FIGURE 2:**
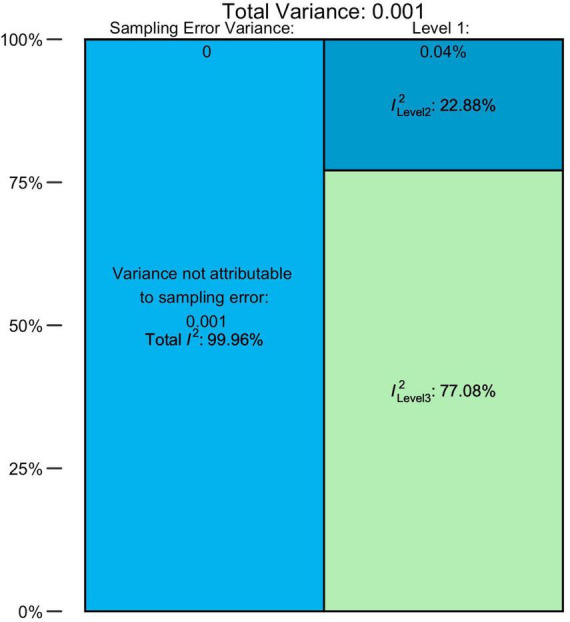
The distribution of the total variance for ASD.

The overall pooled prevalence of ASD (*N* = 79, *n* = 220) from 1996 to 2019 was 0.72% (95% CI = 0.61–0.85). There was an unacceptably high heterogeneity, with a total *I*^2^ = 99.96%. The distribution of variance over the three levels of our model is illustrated in Figure 2 and Supplementary Table 8, indicating that approximately 0.04% of the overall variance can be attributed to Level 1 (sampling error), 22.88% to Level 2 (within-studies), and 77.08% to Level 3 (between-studies).

For AD, AS, and AA and PDD-NOS groups, the pooled prevalence estimates comprised 0.25% (95% CI = 0.18–0.33%), 0.13% (95% CI = 0.07–0.20), and 0.18% (95% CI = 0.10–0.28) accordingly. Heterogeneity was also very high for all these diagnostic groups. Detailed estimations of the heterogeneity variance attributable to different levels of the meta-analysis are presented in Supplementary Table 8. Overall, the sampling error on Level 1 was small and ranged from 1.30 to 2.57%. The values of *I*^2^ on Level 2 were much higher, totaling from 14.19 to 59.73%, with the exclusion of the AS group, for which the within-study heterogeneity remained low (2.34%). In general, the largest share fell to Level 3 (between-study) of heterogeneity, which explained 84.51 and 95.09% of variance for AD and AS groups, respectively. Interestingly, AA/PDD-NOS was behind this trend: for this group, between-study heterogeneity (44.80%) was lower than within-study (53.23%).

### 3.4. Moderator analysis

#### 3.4.1. Prevalence of ASD across different geographical and socio-economical regions

**FIGURE 3:**
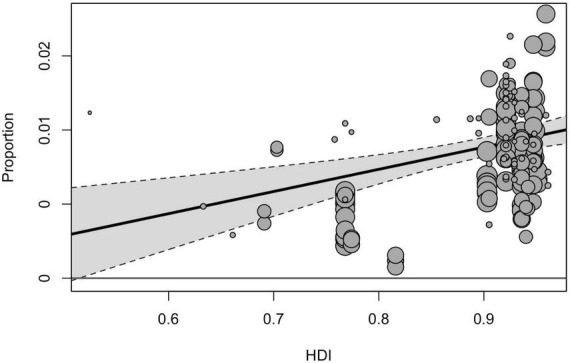
The meta-regression analysis of the effect of the HDI on the pooled prevalence of ASD.

Geographical region, country, and country income variables tested in the moderator analyses yielded a significant effect on prevalence estimates [*F*(2, 211) = 8.58, *p* < 0.001, *F*(9, 173) = 6.65, *p* < 0.001, *F*(3, 214) = 199.60, *p* < 0.001]. Among three geographical regions included in the sub-group analysis, the pooled prevalence of ASD was significantly higher in North America (1.01% [95% CI = 0.79–1.25]), compared with estimates obtained for Europe (0.73% [95% CI = 0.57–0.91], *p* < 0.001) and Asia (0.41% [95% CI = 0.26–0.59], *p* < 0.001). When analyzed by income groups (with the exclusion of low-income countries), the highest prevalence was in high-income countries (0.79% [95% CI = 0.67–0.93]), and the lowest–in lower middle-income ones (0.32% [95% CI = 0.80–0.71]). Based on the country level, the highest prevalence of ASD was reported in the USA (1.12% [95% CI = 0.92–1.33]), followed by Sweden (0.90% [95% CI = 0.59–1.27]) and Denmark (0.73% [95% CI = 0.49–1.04]), the lowest estimates were calculated for Taiwan (0.11% [95% CI = 0.02–0.29]), France (0.32% [95% CI = 0.12–0.61]), and China (0.42% [95% CI = 0.21–0.70]). The total heterogeneity remained substantially high for all analyzed subgroups, and the tested heterogeneity estimates not explained by the predictors were significant for all moderators [*QE*(173) = 48960.02, *p* < 0.001, *QE*(211) = 108119.08, *p* < 0.001, *QE*(214) = 251988.31, *p* < 0.001 for the country, geographical region, and country income variables, respectively]. Univariate meta-regression model accounted for HDI and showed that studies from countries with higher HDI tended to report higher prevalence estimates (Figure 3).

#### 3.4.2. The effect of study time on the prevalence

**FIGURE 4:**
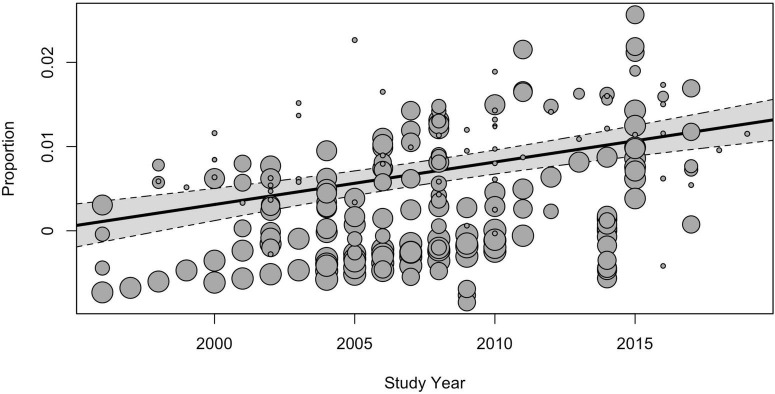
The meta-regression analysis of the effect of the study time on the pooled prevalence of ASD.

The subgroup analysis of moderators showed that the study time influenced the prevalence estimates. The global pooled point-prevalence of ASD consistently increased from 0.25% (95% CI = 0.12–0.42) during the period from 1994 to 1999 to 0.99 (95% CI = 0.73–1.28) in the 2015–2019 period. Despite the clear time trend, the total *I*^2^ indexes for heterogeneity remained unacceptably high, mostly due to the between-study variance, and the remaining heterogeneity not explained by study time was still highly substantial [*QE*(215) = 131,985.52, *p* < 0.001]. Univariate meta-regression model with continuous time variable (Figure 4) also demonstrated the rising prevalence over time [*F*(1, 218) = 35.53, *p* < 0.001], while the model still yielded unacceptably high heterogeneity (*I*^2^_Level2_ = 19.58%, *I*^2^_Level3_ = 80.37%).

#### 3.4.3. The effect of age on the prevalence

The age group moderator also demonstrated a significant effect on the pooled prevalence [*F*(2, 159) = 6.11, *p* < 0.01], while heterogeneity not explained by age group was still significant [*QE*(159) = 109956.86, *p* < 0.001]. The prevalence was significantly higher for children aged between 6 and 12 years (0.82% [95% CI = 0.67–0.98]) compared to the subgroup of children under the age of 5 (0.60% [95% CI = 0.45–0.76]) and those, than older 13 years (0.57% [95% CI = 0.40–0.76]).

#### 3.4.4. The effect of study design and risk of bias

A significant moderating effect was found for the type of study design [*F*(5, 214) = 4.08, *p* < 0.01], while for the risk of bias, it was not revealed [*F*(2, 217) = 0.35, *p* < 0.05]. The heterogeneity not explained by study design and risk of bias remained significant for both tested variables [*QE*(214) = 167,848.72, *p* < 0.001, *QE*(217) = 202,284.97, *p* < 0.001]. Specifically, the lowest estimates were obtained from studies, implemented health insurance (0.35% [95% CI = 0.10–0.77]), and different types of administrative databases (0.48% [95% CI = 0.31–0.68]). Substantially higher estimates were revealed from studies where cases were identified based on records-review surveillance (1.22% [95% CI = 0.91–1.56]), followed by studies with mixed designs (0.80% [95% CI = 0.52–1.14]), direct surveillance (0.75% [95% CI = 0.51–1.03]), and registers (0.66% [95% CI = 0.44–0.93]).

### 3.5. Analysis of outliers

**FIGURE 5:**
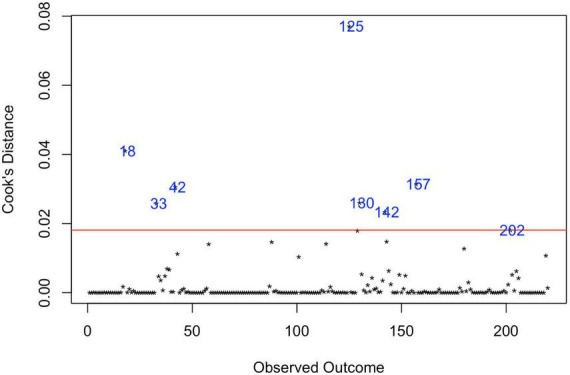
Influential observations by Cooks distance. 18—Hewitt et al. (43); 33—Zahorodny et al. (37); 42—Akhter et al. (44); 125—Kim et al. (47); 130—Aguilera et al. (38); 142—Baio et al. (45); 157—Maenner et al. (46); 202—Shaw et al. (48).

Analysis of Cook’s distance (Figure 5) marked 8 data points (37, 38, 43–48) as the most influential based on the 4/n cut-off of Cook’s distance (49), with the most prominent estimate, obtained in the study of Kim et al. (47). Six data points represent the largest estimates from the original data set, five of them were extracted from USA studies, based on records review surveillance design, that, as was mentioned above, is associated with the largest prevalence estimates compared with other methodologies. Accounting for these red-flag data points, the re-running meta-analysis after removing these marked data-points did not reduce heterogeneity (*I*^2^_Level2_ = 26.98%, *I*^2^_Level3_ = 73.37%, *I*^2^_Total_ = 99.95%).

### 3.6. Recalculating and adjusting pooled prevalence of ASD

Given that the results of moderator analysis indicate that there is a number of different variables that influence pooled prevalence estimate of ASD, while none of them leads to any appreciable decrease in heterogeneity and remarkable significant increase estimates over time, lower estimates in low- and lower income countries and higher in countries from Europe and North America, we decided to calculate prevalence for countries with upper middle and higher income, from Europe and North America, with estimates for 2015–2019, applying age of participants and study design variables as moderators. For the obtained subgroup, the pooled adjusted prevalence comprised 1.18% (95% CI = 0.92–1.48). However, the heterogeneity for this estimate was also still very high due to within studies effects (*I*^2^_Level2_ = 99.75%, *I*^2^_Level3_ = 0%).

## 4. Discussion

Overall, 85 articles with 317 extracted prevalence estimates were included in the quantitative synthesis, covering the period from 1994 to 2019 and a population of 120,130,596 individuals from 29 countries. Compared to previous studies aimed at assessing worldwide prevalence, we have tried to select potentially the most reliable data by including in the analysis only those studies in which case identification was based on formal diagnoses according to ICD and DSM classifications or on the results of so-called “gold standard” methods (ADOS and ADI). Thus, this study lacks the results of a large pool of data from studies based on national surveys and studies that applied diagnoses according to classifications other than ICD and DSM, or those, without information about case identification. Particularly, this study also did not include data from studies in which case identification was based solely on the educational diagnosis of ASD, as in many systems, it is often based on the most appropriate service delivery approach, so children with ASD may be qualified under another disability category, and indeed children without ASD with severe socio-communication impairments could be assigned under ASD for receiving an appropriate educational plan, as in case of classification in the framework of The Individuals with Disabilities Education Act (IDEA) (50).

Among the included studies, a substantial number was presented by high-income countries (83.5%), mainly from the USA (23.5%). Analysis of raw data found that the average percentage of individuals classified as having an intellectual disability in our sample was 34.9 ± 13.5, 60.0 ± 23.8, 3.4 ± 4.5, 24.1 ± 21.6 for ASD, AD, AS, and AA/PDD, respectively. However, it is rather difficult to compare found results with those available in the literature, as they vary considerably, and no one comprehensive review or meta-analysis was not identified.

The mean male-to-female ratio was 4.3 (Med = 4.2, *SD* = 1.5) for ASD and 4.2 (Med = 4.2, *SD* = 1.6) for AD. For other subgroups, the ratio was more variable, and the gender ratio increased to 9.7 (Med = 6.2, *SD* = 8.0) for AS and 7.0 (Med = 4.3, *SD* = 3.0) for AA. For the whole group of ASD, the obtained estimate is equal to the proportions found in the systematic review of the ASD prevalence performed by Zeidan et al. (18) and in the meta-analysis of Loomes et al. (93), focusing specifically on the male-to-female ratio. However, when the low-quality studies were eliminated in the latter meta-analysis, the ratio was decreased to 3.32.

The prevalence estimates, produced by a three-level meta-analysis, comprised 72 per 10,000 (95% CI = 61–85) for ASD, 25 (95% CI = 18–33) for AD, 13 (95% CI = 7–20) for AS, and 18 (95% CI = 10–28) for the combined group of AA and PDD-NOS. However, as expected, a large amount of variation in prevalence across studies was found due to within- and between-study heterogeneity, which substantially reduces the reliability of these estimates.

Univariate analysis of moderators, associated with study location, revealed that pooled prevalence of ASD was significantly higher in North America (1.01% [95% CI = 0.79–1.25]), compared with estimates obtained for Europe (0.73% [95% CI = 0.57–0.91]) and Asia (0.41% [95% CI = 0.26–0.59]), where among countries that were included in the subgroup analysis the estimate from the USA (1.12% [95% CI = 0.92–1.33]) was the highest, while the lowest prevalence was identified in Taiwan (0.11% [95% CI = 0.02–0.29]). Similar results were identified in the systematic review and meta-analysis of ASD prevalence by Salari et al. published in 2022: for America, the prevalence was 1%, and for Asia–0.4%. However, for Europe, the prevalence was lower–0.5%. As in the cited study, the most prominent estimate was obtained for the region of Australia; it should be noted that in our analysis, the whole region of Oceania (along with Africa) was not included in the subgroup analysis due to the lack of data, eligible for this type of analysis.

Our findings also confirm a clear trend associated with country income and HDI: countries with higher income and HDI tend to report higher prevalence estimates. It may be assumed that this difference could be attributed to the detection gap, associated with lower access to appropriate diagnostics, lower awareness among parental and professional communities about autism, and specific cultural attitudes to the developmental and mental disorders that may influence pathways of families to seek care (51). The effect of socio-economic factors on the prevalence of ASD is evident not only in the case of between-countries comparisons but also it is reported for differences between different communities within one study. As an example, a study conducted in Texas found that the state’s high-income community had a six times higher prevalence of ASD than the lowest-income group, as per the administrative data (52). On the other hand, the effects of country income and HDI in our analysis may also be confounded by the fact that high-income countries are much more represented in our sample compared with low-income and are based on larger sample sizes.

As was expected, the subgroup analysis also revealed a significant moderator effect of study time, reported in the scope of previous research: the global pooled point-prevalence of ASD consistently increased from 0.25% (95% CI = 0.12–0.42) during the period from 1994 to 1999 to 0.99 (95% CI = 0.73–1.28) in the 2015–2019 period.

As for age groups, the prevalence of ASD was significantly higher for children aged between 6 and 12 years (0.82% [95% CI = 0.67–0.98]) compared to the subgroup of children under the age of 5 (0.60% [95% CI = 0.45–0.76]) and those, older than 13 years (0.57% [95% CI = 0.40–0.76]). As it is mentioned by Fombonne et al. (10), by ages 6–10, diagnoses can be assigned more robustly, while at lower ages, some children could be missed up to primary school entry or later when social demands from a child increase and specific autistic impairments become more evident. At the same time, at older ages, some improvements in milder forms of ASD can pose challenges for both identification and diagnostic confirmation.

Regarding the moderator analysis for the methodological aspects of studies, it was shown that the lowest estimates were obtained from studies based on information from health insurance (0.35% [95% CI = 0.10–0.77]) and different types of administrative databases (0.48% [95% CI = 0.31–0.68]). Substantially higher estimates were revealed from studies where cases were identified based on records-review surveillance (1.22% [95% CI = 0.91–1.56]), followed by studies with mixed designs (0.80% [95% CI = 0.52–1.14]), direct surveillance (0.75% [95% CI = 0.51–1.03]), and registers (0.66% [95% CI = 0.44–0.93]). These findings are predictable, as they substantially vary in their complexity, reliability, and consistency of instruments used, the professional level of clinicians participating in the case identification, feasible sample sizes, and cost (10, 53). Thus, studies that use existing databases with routinely collected health information have the lowest cost and provide large sample sizes but do not control effects associated with the professional level of clinicians and reliability of diagnostic methods and are unable to count individuals without an official diagnosis, which could underestimate the prevalence of ASD. At the same time, methods based on records-review surveillance also rely on available clinical and educational information obtained in routine practice. However, these records are scrutinized through two phases by trained experts. The case finding phase involves systematic screening of multi-source records by trained record abstractors aimed at revealing the “red flags” of ASD. In the case of screening-positive results, all available information is extracted and subjected to in-depth evaluation by trained clinicians specializing in the diagnostics of ASD (53). So, even if a child does not have an official diagnosis of ASD, he/she could be classified under this category, and new cases could be established. Nevertheless, since this design does not involve a direct assessment of a child and a direct interview with a child’s caregivers, this design could rely on mechanical rules in the interpretation of available records, lacking clinical judgment and, as a result, overestimate prevalence estimates of ASD (54, 55).

Although all the moderating variables examined in this study showed a significant effect on the pooled prevalence estimate, none of them showed a significant effect on reducing remaining heterogeneity. To get more reliable prevalence estimates, we performed additional steps. Based on the assumption that more recent data (in relation to the improvements in the unification of diagnostic criteria of ASD and the development of more precise diagnostic instruments) from middle- and higher-income countries in Europe and North America are presumably more reliable for ASD prevalence (because they probably have a better case identification policy and have more resources for training of clinical experts and access to reliable diagnostic instruments), we narrowed our sample to estimates from 2015 to 2019 and these regions, using age of participants and study design variables as covariates for the final model. Thus, the final prevalence rate was 1.18% (95% CI = 0.92–1.48), which seemed closer to the up-to-date mean prevalence and the consensus estimate of 1%, accepted by the World Health Organization (56). However, the heterogeneity for this estimate was still very high due to within studies effects, so this result should be interpreted with caution.

This study, to our knowledge, is one of the most comprehensive attempts to address the issue of worldwide prevalence of ASD and previously existing relative diagnoses using meta-analytical statistics and the first that employed the three-level approach to deal with multiple estimates from a single study. Inclusion criteria were stricter than other systematic reviews, which could result in capitalizing on more reliable data.

However, there are a number of limitations. First, only those studies published in English were included, so several studies were missed due to language limitations. Second, partly because of limited data in the literature, partly due to the stricter inclusion criteria, the meta-regression analyses might be under-powered (as we applied a minimal 5 data-point cut-off per level of covariate), so obtained results of the source of heterogeneity should be considered as an exploratory and require future confirmation. Finally, very substantial heterogeneity remained unaccounted for across all subgroups and models examined, even when between-study variance was eliminated and more complex models were applied. As mentioned above, this fact substantially limits the reliability and generalizability of the obtained results.

## Data availability statement

The original contributions presented in this study are included in the article/Supplementary material, further inquiries can be directed to the corresponding author.

## Author contributions

OIT and EG developed the concept and design of the study. DK, ES, OIT, OST, PS, RR, and TD with the exclusion of EG, participated in the data collection. OIT and RR wrote the manuscript. All authors contributed to and approved its final version.
