# Supplementary material for: The global prevalence of autism spectrum disorder: A three-level meta-analysis
Source: Front Psychiatry. 2023 Feb 9;14:1071181. doi: 10.3389/fpsyt.2023.1071181 (PMC9947250; doi:10.3389/fpsyt.2023.1071181)
Supplement: Supplementary file 1 [file Data_Sheet_1.docx]

**Supplementary Table 1.** Data for Autism Spectrum Disorder included in the meta-analysis

| **Est. ID** | **Author, Year** | **Country** | **Area(s)** | **Geographical region** | **Income** | **HDI** | **Study time** | **Age, years** | **Design** | **Case criterion** | **Sample Size** | **Sample Size for MA** | **Number of Cases** | **Number of Cases for MA** | **Prevalence per 10000 (95% CI)** | **Gender ratio** | **IQ Score < 70 (%)** | **RoB** |
| --- | --- | --- | --- | --- | --- | --- | --- | --- | --- | --- | --- | --- | --- | --- | --- | --- | --- | --- |
| **1** | Parner, 2011 | Denmark | Denmark | Europe | HI | 0.948 | 2004 | 5-6 | Reg | ICD-10 | 131,842 | 114,356.40 | 462 | 462.00 | 40.0 (36.7–44.4) | 4.6 | NA | 4 |
| **2** | Parner, 2011 | Denmark | Denmark | Europe | HI | 0.948 | 2004 | 7-8 | Reg | ICD-10 | 134,505 | 122,882.90 | 682 | 682.00 | 56.0 (51.3–59.9) | 4.9 | NA | 4 |
| **3** | Parner, 2011 | Denmark | Denmark | Europe | HI | 0.948 | 2004 | 9-10 | Reg | ICD-10 | 138,469 | 130,395.10 | 858 | 858.00 | 66.0 (61.4–70.5) | 5.3 | NA | 4 |
| **7** | Parner, 2011 | Australia | West. Australia | Oceania | HI | 0.951 | 2004 | 5-6 | Reg | DSM-IV(TR) | 50,803 | 49,887.60 | 222 | 222.00 | 45.0 (39.0–50.8) | 4.6 | NA | 4 |
| **8** | Parner, 2011 | Australia | West.  Australia | Oceania | HI | 0.951 | 2004 | 7-8 | Reg | DSM-IV(TR) | 50,125 | 48,560.50 | 253 | 253.00 | 52.0 (46.0–59.0) | 4.3 | NA | 4 |
| **9** | Parner, 2011 | Australia | West. Australia | Oceania | HI | 0.951 | 2004 | 9-10 | Reg | DSM-IV(TR) | 50,120 | 49,391.70 | 203 | 203.00 | 41.0 (35.8–47.2) | 6.3 | NA | 4 |
| **13** | Samadi, 2012 | Iran | Iran | Asia | LMI | 0,774 | 2006 | 5 | AD | ADI-R | 443,333 | 443,33.,00 | 324 | 324.00 | 7.3 (6.5–8.2) | 4.1 | NA | 4 |
| **14** | Samadi, 2012 | Iran | Iran | Asia | LMI | 0.774 | 2007 | 5 | AD | ADI-R | 451,202 | 451,202.00 | 231 | 231.00 | 5.1 (4.9–5.8) | 3.2 | NA | 4 |
| **15** | Samadi, 2012 | Iran | Iran | Asia | LMI | 0.774 | 2008 | 5 | AD | ADI-R | 425,799 | 425,799.00 | 289 | 289.00 | 6.8 (6.1–7.6) | 4.2 | NA | 4 |
| **16** | Hamad. 2019 | Canada | Manitoba | North America | HI | 0.936 | 2005 | 1-5 | Reg | ICD-9/10 | 499,93 | 49,993.00 | 239 | 239.00 | 47.8 (NA) | 3.2 | NA | 5 |
| **17** | Hamad. 2019 | Canada | Manitoba | North America | HI | 0.936 | 2010 | 1-5 | Reg | ICD-9/10 | 550,68 | 55,068.00 | 291 | 291.00 | 52.8 (NA) | 4.3 | NA | 5 |
| **18** | Hamad. 2019 | Canada | Manitoba | North America | HI | 0.936 | 2011 | 1-5 | Reg | ICD-9/10 | 566,68 | 56,668.00 | 316 | 316.00 | 55.8 (NA) | 4.0 | NA | 5 |
| **19** | Hamad. 2019 | Canada | Manitoba | North America | HI | 0.936 | 2012 | 1-5 | Reg | ICD-9/10 | 575,18 | 57,518.00 | 384 | 384.00 | 66.8 (NA) | 3.2 | NA | 5 |
| **20** | Hamad. 2019 | Canada | Manitoba | North America | HI | 0.936 | 2013 | 1-5 | Reg | ICD-9/10 | 581,04 | 58,104.00 | 477 | 477.00 | 82.1 (NA) | 3.4 | NA | 5 |
| **21** | Hamad. 2019 | Canada | Manitoba | North America | HI | 0.936 | 2014 | 1-5 | Reg | ICD-9/10 | 587,65 | 58,765.00 | 514 | 514.00 | 87.5 (NA) | 3.9 | NA | 5 |
| **22** | Hamad. 2019 | Canada | Manitoba | North America | HI | 0.936 | 2015 | 1-5 | Reg | ICD-9/10 | 594,38 | 59,438.00 | 578 | 578.00 | 97.2 (NA) | 4.1 | NA | 5 |
| **23** | Ellefsen, 2007 | Denmark | Faroe Island | Europe | HI | 0.948 | 2002 | 7-16 | Mixed (DS&AD) | ICD-10; Gilberg criteria | 7,689 | 7,689.00 | 41 | 41.00 | 53.3 (36–70) | 6.0 | 31.7 | 2 |
| **27** | Hewitt, 2016 | USA | Minneapolis | North America | HI | 0.921 | 2010 | 7-9 | RRS | DSM-IV-TR | 12,329 | 12,329.00 | 255 | 255.00 | 206.8 (183–234) | 4.2 | 32.6 | 5 |
| **28** | Nicholas, 2009 | USA | South Carolina | North America | HI | 0.921 | 2006 | 4 | RRS | DSM-IV-TR | 8,156 | 8,156.00 | 65 | 65.00 | 79.7 (61–99) | 4.8 | 55.8 | 5 |
| **29** | Idring, 2012 | Sweden | Stockholm County | Europe | HI | 0.947 | 2007 | 4-6 | Reg | ICD-9/10, DSM-IV | 66,571 | 66,571.00 | 434 | 434.00 | 65.2 (59–71) | NA | 64.5 | 4 |
| **30** | Idring, 2012 | Sweden | Stockholm County | Europe | HI | 0.947 | 2007 | 7-12 | Reg | ICD-9/10, DSM-IV | 127,428 | 127,428.00 | 1,524 | 1,524.00 | 119.6 (114–126) | NA | 52.1 | 4 |
| **31** | Idring, 2012 | Sweden | Stockholm County | Europe | HI | 0.947 | 2007 | 13-17 | Reg | ICD-9/10, DSM-IV | 125,271 | 125,271.00 | 1,834 | 1,834.00 | 146.4 (140–153) | NA | 59.1 | 4 |
| **32** | Idring, 2012 | Sweden | Stockholm County | Europe | HI | 0.947 | 2007 | 18-23 | Reg | ICD-9/10, DSM-IV | 124,884 | 124,884.00 | 1,308 | 1,308.00 | 104.7 (99–110) | NA | 59.0 | 4 |
| **33** | Jin, 2018 | China | Shanghai | Asia | UMI | 0.768 | 2014 | 3 | DS | DSM-5 | 2,364 | 2,364.00 | 7 | 6.95 | 29.4 (21.3–37.5) | 6.0 | NA | 3 |
| **34** | Jin, 2018 | China | Shanghai | Asia | UMI | 0.768 | 2014 | 4 | DS | DSM-5 | 9,452 | 9,452.00 | 21 | 15.60 | 16.5 (13.5–19.6) | 4.3 | NA | 3 |
| **35** | Jin, 2018 | China | Shanghai | Asia | UMI | 0.768 | 2014 | 5 | DS | DSM-5 | 9,910 | 9,910.00 | 18 | 6.44 | 6.5 (4.7–8.4) | 3.5 | NA | 3 |
| **36** | Jin, 2018 | China | Shanghai | Asia | UMI | 0.768 | 2014 | 6 | DS | DSM-5 | 9,602 | 9,602.00 | 14 | 4.13 | 4.3 (2.8–5.9) | 6.0 | NA | 3 |
| **37** | Jin, 2018 | China | Shanghai | Asia | UMI | 0.768 | 2014 | 7 | DS | DSM-5 | 9,224 | 9,224.00 | 20 | 3.87 | 4.2 (2.7–5.8) | 3.0 | NA | 3 |
| **38** | Jin, 2018 | China | Shanghai | Asia | UMI | 0.768 | 2014 | 8 | DS | DSM-5 | 9,702 | 9,702.00 | 32 | 7.08 | 7.3 (5.3–9.3) | 2.2 | NA | 3 |
| **39** | Jin, 2018 | China | Shanghai | Asia | UMI | 0.768 | 2014 | 9 | DS | DSM-5 | 8,379 | 8,379.00 | 25 | 5.45 | 6.5 (4.4–8.5) | 4.0 | NA | 3 |
| **40** | Jin, 2018 | China | Shanghai | Asia | UMI | 0.768 | 2014 | 10 | DS | DSM-5 | 7,449 | 7,449.00 | 23 | 5.29 | 7.1 (4.9–9.4) | 2.8 | NA | 3 |
| **41** | Jin, 2018 | China | Shanghai | Asia | UMI | 0.768 | 2014 | 11 | DS | DSM-5 | 5,815 | 5,815.00 | 27 | 5.64 | 9.7 (6.7–12.7) | 4.4 | NA | 3 |
| **42** | Zahorodny, 2014 | USA | New Jersey | North America | HI | 0.921 | 2006 | 8 | RRS | DSM-IV-TR | 30,570 | 30,570.00 | 533 | 533.00 | 174.4 (159–189) | 5.1 | 23.3 | 5 |
| **44** | Baio, 2012 | USA | 14 ADDM sites | North America | HI | 0.921 | 2008 | 8 | RRS | DSM-IV-TR | 337,093 | 337,093.00 | 3,820 | 3,820.00 | 113.3 (110–117) | 4.8 | 38.0 | 5 |
| **45** | Windham, 2011 | USA | San Francisco Bay Area | North America | HI | 0.921 | 2002 | 8 | RRS | DSM-IV | 82,153 | 82,153.00 | 383 | 383.00 | 46.6 (42–51) | 6.1 | NA | 6 |
| **46** | Rice, 2009 | USA | 11 ADDM sites | North America | HI | 0.921 | 2006 | 8 | RRS | DSM-IV-TR | 308,038 | 308,038.00 | 2,757 | 2,757.00 | 89.5 (86–93) | 4.8 | 41.0 | 5 |
| **51** | Narzisi, 2020 | Italy | Pisa | Europe | HI | 0.895 | 2016 | 7-9 | Mixed (DS&RRS) | DSM-5 | 4,417 | 4,417.00 | 50,7 | 50.70 | 114.8 (83–146) | NA | NA | 3 |
| **52** | Diallo, 2018 | Canada | Quebec | North America | HI | 0.936 | 2014 | 1-17 | AD | ICD-9/10 | 1,464,600 | 1,464,600.00 | 16,940 | 17,868.00 | 122.0 (NA) | NA | NA | 7 |
| **53** | Pelly, 2015 | Canada | Avalon Peninsula | North America | HI | 0.936 | 2010 | 4 | AD | DSM-IV-TR | 2,667.5 | 2,667.50 | 33 | 33.00 | 123.7 (88.5–178.6) | 10.0 | NA | 4 |
| **54** | CDC ADDM, 2007 | USA | 6 ADDM sites | North America | HI | 0.921 | 2000 | 8 | RRS | DSM-IV-TR | 187,761 | 18,7761.00 | 1252 | 1,252.00 | 66.7 (45–99) | 3.7 | NA | 6 |
| **55** | CDC ADDM, 2007 | USA | 14 ADDM sites | North America | HI | 0.921 | 2002 | 8 | RRS | DSM-IV-TR | 407,578 | 407,578.00 | 2,685 | 2,685.00 | 65.8 (63–68) | 4.4 | 44.6 | 5 |
| **56** | Akhter, 2018 | Bangladesh | Raiganj | Asia | LMI | 0.661 | 2016 | 1,5-2 | DS | DSM-IV-TR | 5,286 | 5,286.00 | 4 | 4.00 | 7.6 (NA) | 3.0 | NA | 3 |
| **57** | Huang, 2014 | China | Tianjin | Asia | UMI | 0.768 | 2009 | 1,5-2 | DS | DSM-IV | 8,000 | 8,000.00 | 22 | 22.00 | 27.5 (16–39) | 4.5 | NA | 3 |
| **58** | Montiel-Nava, 2008 | Venezuela | Maracaibo | South America | NA | 0.691 | 2005 | 3-5 | AD | DSM-IV-TR | 122,877 | 122,877.00 | 250 | 250.00 | 20.3 (15–27) | 2.9 | NA | 3 |
| **60** | Montiel-Nava, 2008 | Venezuela | Maracaibo | South America | NA | 0.691 | 2005 | 6-9 | AD | DSM-IV-TR | 132,028 | 132,028.00 | 180 | 180.00 | 13.6 (12–15.7) | 3.9 | NA | 3 |
| **62** | Bachmann, 2018 | Germany | Germany | Europe | HI | 0.942 | 2006 | 0-24 | HID | ICD-10 | 6,900,000 | 6,704,090.90 | 14,749 | 14,749.00 | 22.0 (NA) | 2.1 | NA | 5 |
| **63** | Bachmann, 2018 | Germany | Germany | Europe | HI | 0.942 | 2012 | 0-24 | HID | ICD-10 | 6,400,000 | 5,601,578.90 | 21,286 | 21,186.00 | 38.0 (NA) | 2.8 | NA | 5 |
| **64** | Tebruegge, 2004 | UK | Kent | Europe | HI | 0.929 | 2000 | 8-9 | AD | ICD-10 | 2,536 | 2,536.00 | 21 | 21.00 | 82.8 (NA) | 6.0 | NA | 3 |
| **67** | van Bakel, 2015 | France | Haute-Garonne, Isère, Savoy, Upper-Savoy | Europe | HI | 0.903 | 2004 | 7 | Reg | ICD-10 | 42,360 | 42,360.00 | 111 | 111.00 | 26.2 (21.6–31.5) | NA | NA | 4 |
| **70** | van Bakel, 2015 | France | Haute-Garonne, Isère, Savoy, Upper-Savoy | Europe | HI | 0.903 | 2005 | 7 | Reg | ICD-10 | 42,724 | 42,724.00 | 145 | 145.00 | 33.9 (28.6–40) | NA | NA | 4 |
| **73** | van Bakel, 2015 | France | Haute-Garonne, Isère, Savoy, Upper-Savoy | Europe | HI | 0.903 | 2006 | 7 | Reg | ICD-10 | 43,977 | 43,977.00 | 144 | 144.00 | 32.7 (27.6–38.5) | NA | NA | 4 |
| **76** | van Bakel, 2015 | France | Haute-Garonne, Isère, Savoy, Upper-Savoy | Europe | HI | 0.903 | 2007 | 7 | Reg | ICD-10 | 44,275 | 44,275.00 | 172 | 172.00 | 38.8 (33.3–45.1) | NA | NA | 4 |
| **79** | van Bakel, 2015 | France | Haute-Garonne, Isère, Savoy, Upper-Savoy | Europe | HI | 0.903 | 2008 | 7 | Reg | ICD-10 | 44,645 | 44,645.00 | 184 | 184.00 | 41.2 (35.5–47.6) | NA | NA | 4 |
| **82** | van Bakel, 2015 | France | Haute-Garonne, Isère, Savoy, Upper-Savoy | Europe | HI | 0.903 | 2009 | 7 | Reg | ICD-10 | 44,742 | 44,742.00 | 181 | 181.00 | 40.5 (34.8–46.8) | NA | NA | 4 |
| **85** | van Bakel, 2015 | France | Haute-Garonne, Isère, Savoy, Upper-Savoy | Europe | HI | 0.903 | 2010 | 7 | Reg | ICD-10 | 45,028 | 45,028.00 | 186 | 186.00 | 41.3 (35.6–47.7) | NA | NA | 4 |
| **88** | Williams, 2008 | UK | Avon | Europe | HI | 0.929 | 2003 | 11 | AD | ICD-10, SEC | 14,062 | 13,898.00 | 86 | 86.00 | 61.9 (48.8–74.9) | 6,8 | NA | 6 |
| **92** | Kočovská, 2012 | Denmark | Faroe Islands | Europe | HI | 0.948 | 2009 | 15-24 | Mixed (DS&RRS) | ICD-10, DSM-IV, Billstedt et al., 2005 criteria, Gilberg criteria | 7,128 | 7,128.00 | 67 | 67.00 | 94.0 (73–119) | 2.7 | 25.4 | 2 |
| **96** | Christensen, 2018 | USA | 10 ADDM sites | North America | HI | 0.921 | 2012 | 8 | RRS | DSM-IV-TR | 346,978 | 346,978.00 | 5,021 | 5,021.00 | 144.7 (141–149) | 4.7 | 31.4 | 5 |
| **100** | Lai, 2012 | Taiwan | Taiwan | Asia | HI | NA | 2004 | 3-5 | Reg | DSM-III-R/IV(TR) | 846,130 | 846,130.00 | 774 | 774.00 | 9.1 (NA) | NA | NA | 4 |
| **101** | Lai, 2012 | Taiwan | Taiwan | Asia | HI | NA | 2004 | 6-11 | Reg | DSM-III-R/IV(TR) | 1,887,027 | 1,887,027.00 | 2,121 | 2,121.00 | 11.2 (NA) | NA | NA | 4 |
| **102** | Lai, 2012 | Taiwan | Taiwan | Asia | HI | NA | 2004 | 12-14 | Reg | DSM-III-R/IV(TR) | 973,188 | 973,188.00 | 669 | 669.00 | 6.9 (NA) | NA | NA | 4 |
| **103** | Lai, 2012 | Taiwan | Taiwan | Asia | HI | NA | 2004 | 15-17 | Reg | DSM-III-R/IV(TR) | 957,965 | 957,965.00 | 431 | 431.00 | 4.5 (NA) | NA | NA | 4 |
| **104** | Lai, 2012 | Taiwan | Taiwan | Asia | HI | NA | 2005 | 3-5 | Reg | DSM-III-R/IV(TR) | 809,663 | 809,663.00 | 903 | 903.00 | 11.2 (NA) | NA | NA | 4 |
| **105** | Lai, 2012 | Taiwan | Taiwan | Asia | HI | NA | 2005 | 6-11 | Reg | DSM-III-R/IV(TR) | 1,843,489 | 1,843,489.00 | 2,444 | 2,444.00 | 13.3 (NA) | NA | NA | 4 |
| **106** | Lai, 2012 | Taiwan | Taiwan | Asia | HI | NA | 2005 | 12-14 | Reg | DSM-III-R/IV(TR) | 964,802 | 964,802.00 | 762 | 762.00 | 7.9 (NA) | NA | NA | 4 |
| **107** | Lai, 2012 | Taiwan | Taiwan | Asia | HI | NA | 2005 | 15-17 | Reg | DSM-III-R/IV(TR) | 983,879 | 983,879.00 | 575 | 575.00 | 5.8 (NA) | NA | NA | 4 |
| **108** | Lai, 2012 | Taiwan | Taiwan | Asia | HI | NA | 2006 | 3-5 | Reg | DSM-III-R/IV(TR) | 730,819 | 730,819.00 | 957 | 957.00 | 13.1 (NA) | NA | NA | 4 |
| **109** | Lai, 2012 | Taiwan | Taiwan | Asia | HI | NA | 2006 | 6-11 | Reg | DSM-III-R/IV(TR) | 1,826,824 | 1,826,824.00 | 2,795 | 2,795.00 | 15.3 (NA) | NA | NA | 4 |
| **110** | Lai, 2012 | Taiwan | Taiwan | Asia | HI | NA | 2006 | 12-14 | Reg | DSM-III-R/IV(TR) | 968,634 | 968,634.00 | 922 | 92200 | 9.5 (NA) | NA | NA | 4 |
| **111** | Lai, 2012 | Taiwan | Taiwan | Asia | HI | NA | 2006 | 15-17 | Reg | DSM-III-R/IV(TR) | 961,550 | 961,550.00 | 671 | 671.00 | 7.0 (NA) | NA | NA | 4 |
| **112** | Lai, 2012 | Taiwan | Taiwan | Asia | HI | NA | 2007 | 3-5 | Reg | DSM-III-R/IV(TR) | 692,164 | 692,164.00 | 959 | 959.00 | 13.9 (NA) | NA | NA | 4 |
| **113** | Lai, 2012 | Taiwan | Taiwan | Asia | HI | NA | 2007 | 6-11 | Reg | DSM-III-R/IV(TR) | 1,759,057 | 1,759,057.00 | 3,161 | 3,161.00 | 18.0 (NA) | NA | NA | 4 |
| **114** | Lai, 2012 | Taiwan | Taiwan | Asia | HI | NA | 2007 | 12-14 | Reg | DSM-III-R/IV(TR) | 972,584 | 972,584.00 | 1,165 | 1,165.00 | 12.0 (NA) | NA | NA | 4 |
| **115** | Lai, 2012 | Taiwan | Taiwan | Asia | HI | NA | 2007 | 15-17 | Reg | DSM-III-R/IV(TR) | 971,478 | 971,478.00 | 834 | 834.00 | 8.6 (NA) | NA | NA | 4 |
| **116** | Lai, 2012 | Taiwan | Taiwan | Asia | HI | NA | 2008 | 3-5 | Reg | DSM-III-R/IV(TR) | 654,179 | 654,179.00 | 975 | 975.00 | 14.9 (NA) | NA | NA | 4 |
| **117** | Lai, 2012 | Taiwan | Taiwan | Asia | HI | NA | 2008 | 6-11 | Reg | DSM-III-R/IV(TR) | 1682,797 | 1,682,797.00 | 3,420 | 3,420.00 | 20.3 (NA) | NA | NA | 4 |
| **118** | Lai, 2012 | Taiwan | Taiwan | Asia | HI | NA | 2008 | 12-14 | Reg | DSM-III-R/IV(TR) | 968,553 | 968,553.00 | 1,382 | 1,382.00 | 14.3 (NA) | NA | NA | 4 |
| **119** | Lai, 2012 | Taiwan | Taiwan | Asia | HI | NA | 2008 | 15-17 | Reg | DSM-III-R/IV(TR) | 963,101 | 963,101.00 | 994 | 994.00 | 10.3 (NA) | NA | NA | 4 |
| **120** | Lai, 2012 | Taiwan | Taiwan | Asia | HI | NA | 2009 | 3-5 | Reg | DSM-III-R/IV(TR) | 633,676 | 633,676.00 | 1,038 | 1,038.00 | 16.4 (NA) | NA | NA | 4 |
| **121** | Lai, 2012 | Taiwan | Taiwan | Asia | HI | NA | 2009 | 6-11 | Reg | DSM-III-R/IV(TR) | 1,587,433 | 1,587,433.00 | 3,591 | 3,591.00 | 22.6 (NA) | NA | NA | 4 |
| **122** | Lai, 2012 | Taiwan | Taiwan | Asia | HI | NA | 2009 | 12-14 | Reg | DSM-III-R/IV(TR) | 969,690 | 969,690.00 | 1,674 | 1,674.00 | 17.3 (NA) | NA | NA | 4 |
| **123** | Lai, 2012 | Taiwan | Taiwan | Asia | HI | NA | 2009 | 15-17 | Reg | DSM-III-R/IV(TR) | 967,141 | 967,141.00 | 1,176 | 1,176.00 | 12.2 (NA) | NA | NA | 4 |
| **124** | Lai, 2012 | Taiwan | Taiwan | Asia | HI | NA | 2010 | 3-5 | Reg | DSM-III-R/IV(TR) | 621,318 | 621,318.00 | 1,000 | 1,000.00 | 16.1 (NA) | NA | NA | 4 |
| **125** | Lai, 2012 | Taiwan | Taiwan | Asia | HI | NA | 2010 | 6-11 | Reg | DSM-III-R/IV(TR) | 1,538,830 | 1,538,830.00 | 3,893 | 3,893.00 | 25.3 (NA) | NA | NA | 4 |
| **126** | Lai, 2012 | Taiwan | Taiwan | Asia | HI | NA | 2010 | 12-14 | Reg | DSM-III-R/IV(TR) | 912,829 | 912,829.00 | 1,790 | 1,790.00 | 19.6 (NA) | NA | NA | 4 |
| **127** | Lai, 2012 | Taiwan | Taiwan | Asia | HI | NA | 2010 | 15-17 | Reg | DSM-III-R/IV(TR) | 971,456 | 971,456.00 | 1,389 | 1,389.00 | 14.3 (NA) | NA | NA | 4 |
| **128** | Brugha, 2011 | UK | England | Europe | HI | 0.929 | 2007 | ≥16 | DS | ADOS | 7,333 | 7,333.00 | 72 | 72.00 | 98.2 (30–165,0) | NA | NA | 0 |
| **129** | Rudra, 2017 | India | Kolkata | Asia | LMI | 0.633 | 2010 | 3-8 | DS | ADOS | 11,849 | 11,849.00 | 6 | 27.30 | 23.0 (7–46) | NA | NA | 5 |
| **130** | Davidovitch, 2013 | Israel | Israel | Asia | HI | 0.919 | 2010 | 8 | HID | DSM-IV | 37,827 | 37,827.00 | 244 | 244.00 | 64.5 (56.9–73.1) | 4.8 | NA | 3 |
| **131** | Fombonne, 2016 | Mexico | Leon | North America | UMI | 0.758 | 2011 | 8 | Mixed (DS&RRS) | DSM-IV-TR | 12,116 | 12,200.00 | 36 | 106.10 | 87.0 (62–110) | NA | 30.5 | 6 |
| **132** | Chien, 2011 | Taiwan | Taiwan | Asia | HI | NA | 1996 | 0-17 | HID | ICD-9-CM | 268,753 | 268,753.00 | 48 | 48.00 | 1.8 (NA) | NA | NA | 4 |
| **133** | Chien, 2011 | Taiwan | Taiwan | Asia | HI | NA | 1997 | 0-17 | HID | ICD-9-CM | 264,191 | 264,191.00 | 68 | 68.00 | 2.6 (NA) | NA | NA | 4 |
| **134** | Chien, 2011 | Taiwan | Taiwan | Asia | HI | NA | 1998 | 0-17 | HID | ICD-9-CM | 259,255 | 259,255.00 | 102 | 102.00 | 3.9 (NA) | NA | NA | 4 |
| **135** | Chien, 2011 | Taiwan | Taiwan | Asia | HI | NA | 1999 | 0-17 | HID | ICD-9-CM | 253,671 | 253,671.00 | 177 | 177.00 | 7.0 (NA) | NA | NA | 4 |
| **136** | Chien, 2011 | Taiwan | Taiwan | Asia | HI | NA | 2000 | 0-17 | HID | ICD-9-CM | 249,336 | 249,336.00 | 259 | 259.00 | 10.4 (NA) | NA | NA | 4 |
| **137** | Chien, 2011 | Taiwan | Taiwan | Asia | HI | NA | 2001 | 0-17 | HID | ICD-9-CM | 245,666 | 245,666.00 | 358 | 358.00 | 14.6 (NA) | NA | NA | 4 |
| **138** | Chien, 2011 | Taiwan | Taiwan | Asia | HI | NA | 2002 | 0-17 | HID | ICD-9-CM | 241,252 | 241,252.00 | 429 | 429.00 | 17.8 (NA) | NA | NA | 4 |
| **139** | Chien, 2011 | Taiwan | Taiwan | Asia | HI | NA | 2003 | 0-17 | HID | ICD-9-CM | 237,361 | 237,361.00 | 486 | 486.00 | 20.5 (NA) | NA | NA | 4 |
| **140** | Chien, 2011 | Taiwan | Taiwan | Asia | HI | NA | 2004 | 0-17 | HID | ICD-9-CM | 233,365 | 233,365.00 | 565 | 565.00 | 24.2 (NA) | NA | NA | 4 |
| **141** | Chien, 2011 | Taiwan | Taiwan | Asia | HI | NA | 2005 | 0-17 | HID | ICD-9-CM | 229,454 | 229,454.00 | 659 | 659.00 | 28.7 (NA) | NA | NA | 4 |
| **142** | Christensen, 2016 | USA | 5 ADDM sites | North America | HI | 0.921 | 2010 | 4 | RRS | DSM-IV-TR | 58,467 | 58,467.00 | 783 | 783.00 | 133.9 (125-144) | 3.5 | 46.0 | 5 |
| **143** | Lazoff, 2010 | Canada | Montreal | North America | HI | 0.936 | 2008 | 6 | AD | SEC, DSM-IV | 1,627 | 1,627.00 | 21 | 21.00 | 129.1 (74.2-183.9) | NA | NA | 6 |
| **144** | Lazoff, 2010 | Canada | Montreal | North America | HI | 0.936 | 2008 | 7 | AD | SEC, DSM-IV | 1,748 | 1,748.00 | 25 | 25.00 | 143.0 (87.4-198.7) | NA | NA | 6 |
| **145** | Lazoff, 2010 | Canada | Montreal | North America | HI | 0.936 | 2008 | 8 | AD | SEC, DSM-IV | 1,736 | 1,736.00 | 26 | 26.00 | 149.8 (92.6-206.9) | NA | NA | 6 |
| **146** | Lazoff, 2010 | Canada | Montreal | North America | HI | 0.936 | 2008 | 9 | AD | SEC, DSM-IV | 1,769 | 1,769.00 | 23 | 23.00 | 130.0 (77.2-182.8) | NA | NA | 6 |
| **147** | Lazoff, 2010 | Canada | Montreal | North America | HI | 0.936 | 2008 | 10 | AD | SEC, DSM-IV | 1,888 | 1,888.00 | 15 | 15.00 | 79.4 (39.4-119.5) | NA | NA | 6 |
| **148** | Lazoff, 2010 | Canada | Montreal | North America | HI | 0.936 | 2008 | 11 | AD | SEC, DSM-IV | 1,987 | 1,987.00 | 17 | 17.00 | 85.6 (45.1–126.1) | NA | NA | 6 |
| **149** | Lazoff, 2010 | Canada | Montreal | North America | HI | 0.936 | 2008 | 12 | AD | SEC, DSM-IV | 2,169 | 2,169.00 | 19 | 19.00 | 87,6 (48,4–126,8) | NA | NA | 6 |
| **150** | Lazoff, 2010 | Canada | Montreal | North America | HI | 0.936 | 2008 | 13 | AD | SEC, DSM-IV | 2,222 | 2,222.00 | 13 | 13.00 | 58.5 (26.8–90.2) | NA | NA | 6 |
| **151** | Lazoff, 2010 | Canada | Montreal | North America | HI | 0.936 | 2008 | 14 | AD | SEC, DSM-IV | 2,062 | 2,062.00 | 9 | 9.00 | 43.6 (15.2–72.1) | NA | NA | 6 |
| **152** | Lazoff, 2010 | Canada | Montreal | North America | HI | 0.936 | 2008 | 15 | AD | SEC, DSM-IV | 2,282 | 2,282.00 | 11 | 11.00 | 48.2 (19.8–76.6) | NA | NA | 6 |
| **153** | Lazoff, 2010 | Canada | Montreal | North America | HI | 0.936 | 2008 | 16 | AD | SEC, DSM-IV | 2,173 | 2,173.00 | 3 | 3.00 | 13.8 (3–40) | NA | NA | 6 |
| **154** | Lazoff, 2010 | Canada | Montreal | North America | HI | 0.936 | 2008 | 17 | AD | SEC, DSM-IV | 1,972 | 1,972.00 | 5 | 5.00 | 25.4 (8–59) | NA | NA | 6 |
| **155** | Idring, 2015 | Sweden | Stockholm | Europe | HI | 0.947 | 2011 | 0-5 | Reg | ICD-9/10, DSM-IV | 179,655 | 179,655.00 | 712 | 712.00 | 39.6 (37–43) | NA | 17.4 | 6 |
| **156** | Idring, 2015 | Sweden | Stockholm | Europe | HI | 0.947 | 2011 | 6-12 | Reg | ICD-9/10, DSM-IV | 190,512 | 190,512.00 | 3,313 | 3,313.00 | 173.9 (168–180) | NA | 22.1 | 6 |
| **157** | Idring, 2015 | Sweden | Stockholm | Europe | HI | 0.947 | 2011 | 13-17 | Reg | ICD-9/10, DSM-IV | 125,687 | 125,687.00 | 3,096 | 3,096.00 | 246.3 (238–255) | NA | 26.1 | 6 |
| **158** | Idring, 2015 | Sweden | Stockholm | Europe | HI | 0.947 | 2011 | 18-27 | Reg | ICD-9/10, DSM-IV | 239,242 | 239,242.00 | 4,209 | 4,209.00 | 175.9 (171–181) | NA | 29.4 | 6 |
| **161** | Pinborough-Zimmerman, 2012 | USA | Utah | North America | HI | 0.921 | 2002 | 8 | AD | ICD-9 | 26,213 | 26,213.00 | 171 | 171.00 | 65.2 (55–75) | 4.9 | NA | 7 |
| **162** | Pinborough-Zimmerman, 2012 | USA | Utah | North America | HI | 0.921 | 2006 | 8 | AD | ICD-9 | 29,494 | 29,494.00 | 301 | 301.00 | 102.1 (91–113) | 5.3 | NA | 7 |
| **163** | Pinborough-Zimmerman, 2012 | USA | Utah | North America | HI | 0.921 | 2008 | 8 | AD | ICD-9 | 33,757 | 33,757.00 | 432 | 432.00 | 128.0 (118–142) | 4.0 | NA | 7 |
| **164** | Pinborough-Zimmerman, 2012 | USA | Utah | North America | HI | 0.921 | 2006 | 6 | AD | ICD-9 | 32,801 | 32,801.00 | 322 | 322.00 | 98.2 (87–109) | 3.5 | NA | 7 |
| **165** | Pinborough-Zimmerman, 2012 | USA | Utah | North America | HI | 0.921 | 2008 | 6 | AD | ICD-9 | 34,368 | 34,368.00 | 418 | 418.00 | 121.6 (110–134) | 4.5 | NA | 7 |
| **166** | Pinborough-Zimmerman, 2012 | USA | Utah | North America | HI | 0.921 | 2006 | 4 | AD | ICD-9 | 33,955 | 33,955.00 | 256 | 256.00 | 75.4 (67–85) | 4.7 | NA | 7 |
| **167** | Pinborough-Zimmerman, 2012 | USA | Utah | North America | HI | 0.921 | 2008 | 4 | AD | ICD-9 | 35,803 | 35,803.00 | 293 | 293.00 | 81.8 (73–91) | 3.6 | NA | 7 |
| **168** | Kim, 2011 | South Korea | Ilsan district, Goyang | Asia | HI | 0.925 | 2005 | 7-12 | DS | DSM-IV | 23,337 | 55,266.00 | 201 | 1,459.00 | 264.0 (191–337) | 2.7 | NA | 3 |
| **170** | Al-Farsi, 2011 | Oman | Oman | Asia | HI | 0.816 | 2009 | 0-4 | AD | DSM-IV-TR | 270,578 | 270,578.00 | 15 | 15.00 | 0.6 (0.3–0.9) | 14.0 | NA | 4 |
| **171** | Al-Farsi, 2011 | Oman | Oman | Asia | HI | 0.816 | 2009 | 5-9 | AD | DSM-IV-TR | 257,140 | 257,140.00 | 61 | 61.00 | 2.4 (1.9–2.9) | 2.6 | NA | 4 |
| **172** | Al-Farsi, 2011 | Oman | Oman | Asia | HI | 0.816 | 2009 | 10-14 | AD | DSM-IV-TR | 271,195 | 271,195.00 | 37 | 37.00 | 1.4 (0.1–1.8) | 2.4 | NA | 4 |
| **173** | Baron-Cohen, 2009 | UK | Cambridgeshire | Europe | HI | 0.929 | 2003 | 5-9 | Mixed (DS&AD) | ICD-10 | 8,824 | 88,24.00 | 83 | 138.50 | 157 (99–246) | NA | NA | 4 |
| **174** | Aguilera, 2007 | Spain | Seville | Europe | HI | 0.905 | 2002 | 3-21 | AD | DSM-IV | 127,350 | 127,350.00 | 165 | 165.00 | 13.0 (NA) | 4.9 | NA | 4 |
| **178** | Baird, 2006 | UK | South Thames | Europe | HI | 0.929 | 2000 | 9-10 | DS | ICD-10 | 56,946 | 56,946.00 | 158 | 661.10 | 116.1 (90.4–141.8) | 3.3 | 55.0 | 4 |
| **179** | Gillberg, 2006 | Sweden | Gothenburg | Europe | HI | 0.947 | 2001 | 7-12 | Mixed (Reg&RRS) | DSM-IV, Gillberg criteria | 32,568 | 32,568.00 | 262 | 262.00 | 80.4 (70.6–90.3) | 3.6 | NA | 5 |
| **182** | Gillberg, 2006 | Sweden | Gothenburg | Europe | HI | 0.947 | 2001 | 13-18 | Mixed (Reg&RRS) | DSM-IV, Gillberg criteria | 28,507 | 28,507.00 | 175 | 175.00 | 61.4 (NA) | 4.1 | NA | 5 |
| **185** | Gillberg, 2006 | Sweden | Gothenburg | Europe | HI | 0.947 | 2001 | 19-24 | Mixed (Reg&RRS) | DSM-IV, Gillberg criteria | 41,410 | 41,410.00 | 109 | 109.00 | 26.3 (NA) | 3.0 | NA | 5 |
| **192** | Fombonne, 2006 | Canada | Montreal | North America | HI | 0.936 | 2003 | 5-17 | AD | DSM-IV | 27,749 | 27,749.00 | 180 | 180.00 | 64.9 (55.8–75) | 4.8 | NA | 6 |
| **200** | Harrison, 2006 | UK | Scotland, Lothian | Europe | HI | 0.929 | 2001 | 2,5-15 | AD | ICD-10, DSM-IV | 134,661 | 134,661.00 | 443 | 595.70 | 44.2 (39.5–48.9) | 7.0 | NA | 4 |
| **201** | Chakrabarti, 2005 | UK | Stafford, Cannock | Europe | HI | 0.929 | 2002 | 4-6 | DS | DSM-IV | 10,903 | 10,903.00 | 64 | 64.00 | 58.7 (45.2–74.9) | 6.1 | 29.8 | 2 |
| **205** | Scott, 2002 | UK | Cambridgeshire | Europe | HI | 0.929 | 1999 | 5-11 | AD | ICD-10, DSM-IV | 34,262 | 34,262.00 | 196 | 196.00 | 57.2 (49.5–65.8) | 4.0 | NA | 4 |
| **206** | Bertrand, 2001 | USA | Brick Township, New Jersey | North America | HI | 0.921 | 1998 | 3-5 | Mixed (DS&RRS) | DSM-IV | 3,479 | 3,479.00 | 27 | 27.00 | 77.6 (51–113) | NA | NA | 4 |
| **208** | Bertrand, 2001 | USA | Brick Township, New Jersey | North America | HI | 0.921 | 1998 | 6-10 | Mixed (DS&RRS) | DSM-IV | 5,417 | 5,417.00 | 33 | 33.00 | 60.9 (42–85) | NA | NA | 4 |
| **210** | Sun, 2019 | China | Jilin | Asia | UMI | 0.768 | 2013 | 6-10 | DS | DSM-IV-TR/V | 7,258 | 7,258.00 | 77 | 78.40 | 108.0 (87–135) | NA | NA | 5 |
| **211** | Baio, 2018 | USA | 11 ADDM  sites | North America | HI | 0.921 | 2014 | 8 | RRS | DSM-IV-TR | 325,483 | 325,483.00 | 5,473 | 5,473.00 | 168.2 (164–173) | 4.2 | 31.0 | 5 |
| **212** | CDC ADDM, 2014 | USA | 11 ADDM  sites | North America | HI | 0.921 | 2010 | 8 | RRS | DSM-IV-TR | 363,749 | 363,749.00 | 5,338 | 5,338.00 | 146.7 (143–151) | 4.6 | 31.0 | 5 |
| **213** | Saemundsen, 2013 | Iceland | Iceland | Europe | HI | 0.959 | 2009 | 11-15 | Reg | ICD-10 | 22,229 | 22,229.00 | 267 | 267.00 | 120.1 (106.6–135.3) | 2.8 | 45.3 | 0 |
| **217** | Isaksen, 2012 | Norway | Oppland, Hedmark | Europe | HI | 0.961 | 2008 | 6-12 | Mixed (Reg&RRS) | ICD-10 | 31,015 | 31,015.00 | 158 | 158.00 | 50.9 (43–59) | 4,3 | NA | 3 |
| **221** | Parner, 2008 | Denmark | Denmark | Europe | HI | 0.948 | 2006 | 7-8 | Reg | ICD-10 | 132,607 | 115,347.30 | 714 | 714.00 | 61.9 (57.1–67) | NA | NA | 4 |
| **223** | Parner, 2008 | Denmark | Denmark | Europe | HI | 0.948 | 2006 | 9-10 | Reg | ICD-10 | 135,413 | 120,135.10 | 889 | 889.00 | 74.0 (68.3–80.2) | NA | NA | 4 |
| **225** | Parner, 2008 | Denmark | Denmark | Europe | HI | 0.948 | 2006 | 11-12 | Reg | ICD-10 | 139,438 | 127,561.00 | 1,046 | 1,046.00 | 82.0 (76.5–87.8) | NA | NA | 4 |
| **227** | Thomaidis, 2020 | Greece | Greece | Europe | HI | 0.887 | 2019 | 10-11 | AD | ICD-10,  DSM-5 | 182,879 | 182,879.00 | 2,108 | 2,108.00 | 115.3 (114–117) | 4.4 | NA | 3 |
| **228** | CDC ADDM, 2019 | USA | 5 ADDM sites | North America | HI | 0.921 | 2012 | 4 | RRS | DSM-IV-TR | 59,456 | 59,456.00 | 907 | 907.00 | 152.5 (143–163) | 3.9 | 43.6 | 5 |
| **229** | CDC ADDM, 2019 | USA | 6 ADDM sites | North America | HI | 0.921 | 2014 | 4 | RRS | DSM-IV-TR | 70,887 | 70,887.00 | 1,208 | 1,208.00 | 170.4 (161–180) | 4.1 | 46.1 | 5 |
| **230** | Alshaban, 2019 | Qatar | Qatar | Asia | HI | 0.855 | 2015 | 6-11 | Mixed (DS&AD) | DSM-5 | 133,781 | 133,781.00 | 1,099 | 1525.10 | 114 (89–146) | NA | NA | 3 |
| **231** | Saito, 2020 | Japan | Hirosaki | Asia | HI | 0.925 | 2013 | 5 | DS | DSM-5 | 1,310 | 1,310.00 | 22 | 22.00 | 167.9 (98–238) | 1.8 | NA | 2 |
| **232** | Saito, 2020 | Japan | Hirosaki | Asia | HI | 0.925 | 2014 | 5 | DS | DSM-5 | 1,261 | 1,261.00 | 20 | 20.00 | 158.6 (90–228) | 1.9 | NA | 2 |
| **233** | Saito, 2020 | Japan | Hirosaki | Asia | HI | 0.925 | 2015 | 5 | DS | DSM-5 | 1,221 | 1,221.00 | 25 | 25.00 | 204.8 (125–284) | 2,6 | NA | 2 |
| **234** | Saito, 2020 | Japan | Hirosaki | Asia | HI | 0.925 | 2016 | 5 | DS | DSM-5 | 1,224 | 1,224.00 | 20 | 20.00 | 163.4 (92–234) | 3.0 | NA | 2 |
| **235** | Maenner, 2020 | USA | 11 ADDM  sites | North America | HI | 0.921 | 2016 | 8 | RRS | DSM-IV-TR/V | 275,419 | 275,419.00 | 5,108 | 5,108.00 | 185.5 (180–191) | 4.5 | 33.4 | 5 |
| **236** | Delobel-Ayoub, 2020 | Denmark | Denmark | Europe | HI | 0.948 | 2015 | 7 | Reg | ICD-10 | 65,382 | 65,382.00 | 644 | 644.00 | 98.5 (117–135) | 3.7 | 14.1 | 2 |
| **237** | Delobel-Ayoub, 2020 | Denmark | Denmark | Europe | HI | 0.948 | 2015 | 8 | Reg | ICD-10 | 64,550 | 64,550.00 | 811 | 811.00 | 125.6 (91–106) | 3.9 | 12.0 | 2 |
| **238** | Delobel-Ayoub, 2020 | Denmark | Denmark | Europe | HI | 0.948 | 2015 | 9 | Reg | ICD-10 | 65,361 | 65,361.00 | 959 | 959.00 | 146.7 (138–156) | 4.2 | 8.1 | 2 |
| **239** | Delobel-Ayoub, 2020 | Finland | Finland | Europe | HI | 0.940 | 2015 | 7 | Reg | ICD-10 | 59,607 | 59,607.00 | 389 | 389.00 | 65.3 (59–72) | 3.8 | 20.8 | 1 |
| **240** | Delobel-Ayoub, 2020 | Finland | Finland | Europe | HI | 0.940 | 2015 | 8 | Reg | ICD-10 | 58,727 | 58,727.00 | 451 | 451.00 | 76.8 (70–84) | 3.3 | 20.2 | 1 |
| **241** | Delobel-Ayoub, 2020 | Finland | Finland | Europe | HI | 0.940 | 2015 | 9 | Reg | ICD-10 | 58,859 | 58,859.00 | 507 | 507.00 | 86.1 (79–94) | 4.7 | 12.0 | 1 |
| **242** | Delobel-Ayoub, 2020 | Iceland | Finland | Europe | HI | 0.959 | 2015 | 7 | Reg | ICD-10 | 4,625 | 4,625,00 | 111 | 111.00 | 240 (198–288) | 5.2 | 18.9 | 0 |
| **243** | Delobel-Ayoub, 2020 | Iceland | Finland | Europe | HI | 0.959 | 2015 | 8 | Reg | ICD-10 | 4,500 | 4,500.00 | 141 | 141.00 | 313.3 (264–368) | 4.4 | 24.1 | 0 |
| **244** | Delobel-Ayoub, 2020 | Iceland | Finland | Europe | HI | 0.959 | 2015 | 9 | Reg | ICD-10 | 4,426 | 4,426.00 | 111 | 111.00 | 250.8 (207–301) | 3.8 | 18.9 | 0 |
| **245** | Delobel-Ayoub, 2020 | France | Haute-Garonne | Europe | HI | 0.903 | 2015 | 8 | Reg | ICD-10 | 15,836 | 15,836.00 | 115 | 115.00 | 72.6 (60–87) | 5.4 | 38.9 | 2 |
| **246** | Delobel-Ayoub, 2020 | France | Isère, Savoie, Haute-Savoie | Europe | HI | 0.903 | 2015 | 8 | Reg | ICD-10 | 32,342 | 32,342.00 | 154 | 154.00 | 47.6 (40–56) | 4.0 | 24.0 | 2 |
| **247** | Zhou, 2020 | China | 8 cities | Asia | UMI | 0.768 | 2014 | 6 | Mixed (DS&AD) | DSM-5 | 15,070 | 15,070.00 | 43 | 43.00 | 28.5 (20–37) | NA | NA | 2 |
| **248** | Zhou, 2020 | China | 8 cities | Asia | UMI | 0.768 | 2014 | 7 | Mixed (DS&AD) | DSM-5 | 21,574 | 21,574.00 | 69 | 69.00 | 32 (24–40) | NA | NA | 2 |
| **249** | Zhou, 2020 | China | 8 cities | Asia | UMI | 0.768 | 2014 | 8 | Mixed (DS&AD) | DSM-5 | 17,796 | 17,796.00 | 61 | 61.00 | 34.3 (26–43) | NA | NA | 2 |
| **250** | Zhou, 2020 | China | 8 cities | Asia | UMI | 0.768 | 2014 | 9 | Mixed (DS&AD) | DSM-5 | 17,759 | 17,759.00 | 44 | 44.00 | 24.8 (17–32) | NA | NA | 2 |
| **251** | Zhou, 2020 | China | 8 cities | Asia | UMI | 0.768 | 2014 | 10 | Mixed (DS&AD) | DSM-5 | 19,475 | 19,475.00 | 65 | 65.00 | 33.4 (25–41) | NA | NA | 2 |
| **252** | Zhou, 2020 | China | 8 cities | Asia | UMI | 0.768 | 2014 | 11 | Mixed (DS&AD) | DSM-5 | 16,891 | 16,891.00 | 36 | 36.00 | 21.3 (14–28) | NA | NA | 2 |
| **253** | Zhou, 2020 | China | 8 cities | Asia | UMI | 0.768 | 2014 | 12 | Mixed (DS&AD) | DSM-5 | 17,241 | 17,241.00 | 45 | 45.00 | 26.1 (18–34) | NA | NA | 2 |
| **254** | Le, 2019 | Vietnam | Hanoi, Thai Binh, Hoa Binh | Asia | UMI | 0.703 | 2017 | 1,5-1,9 | DS | DSM-IV | 7,128 | 7,128.00 | 55 | 55.00 | 77.2 (58.2–100.3) | NA | NA | 4 |
| **255** | Le, 2019 | Vietnam | Hanoi, Thai Binh, Hoa Binh | Asia | UMI | 0.703 | 2017 | 2-2,5 | DS | DSM-IV | 10,149 | 10,149.00 | 75 | 75.00 | 73.9 (58.2–92.5) | NA | NA | 4 |
| **256** | Valenti, 2019 | Italy | L’Aquila | Europe | HI | 0.895 | 2018 | 3-17 | Reg | DSM-IV/5 | 36,938 | 36,938.00 | 352 | 352.00 | 95.3 (NA) | 4.3 | NA | 4 |
| **257** | Magen-Molho, 2020 | Israel | Israel | Asia | HI | 0.919 | 2016 | 5-16 | HID | DSM-IV-TR/5 | 1,786,194 | 1,786,194.00 | 11,699 | 11,699.00 | 65.5 (NA) | NA | NA | 3 |
| **258** | Pérez‐Crespo, 2019 | Spain | Catalonia | Europe | HI | 0.905 | 2017 | 2-5 | Reg | ICD-9 | 301,153.2 | 301,153.20 | 869 | 869.00 | 28.9 (26–32) | NA | NA | 6 |
| **259** | Pérez‐Crespo, 2019 | Spain | Catalonia | Europe | HI | 0.905 | 2017 | 6-10 | Reg | ICD-9 | 419,226.5 | 419,226.50 | 4,937 | 4,937.00 | 117.8 (114–121) | NA | NA | 6 |
| **260** | Pérez‐Crespo, 2019 | Spain | Catalonia | Europe | HI | 0.905 | 2017 | 11-17 | Reg | ICD-9 | 606,286.4 | 606,286.40 | 9,660 | 10,913.16 | 180 (176–183) | NA | NA | 6 |
| **261** | Fuentes, 2020 | Spain | Basque Country | Europe | HI | 0.905 | 2017 | 7-9 | DS | DSM-IV/5 | 14,734 | 14,734.00 | 87 | 87.00 | 59.0 (48–73) | 6.2 | 41.5 | 4 |
| **262** | Nygren, 2012 | Sweden | Gothenburg | Europe | HI | 0.947 | 2010 | 2 | AD | DSM-IV-TR | 5,007 | 5,007.00 | 40 | 40.00 | 79.9 (57–109) | 4.0 | 35.0 | 2 |
| **265** | Ouellette-Kuntz, 2006 | Canada | Manitoba | North America | HI | 0.936 | 2002 | 1-4 | AD | DSM-IV | 52,280 | 52,280.00 | 109 | 109.00 | 20.8 (17.1–25.1) | NA | NA | 5 |
| **266** | Ouellette-Kuntz, 2006 | Canada | Manitoba | North America | HI | 0.936 | 2002 | 5-9 | AD | DSM-IV | 73,139 | 73,139.00 | 280 | 280.00 | 38.3 (33.9–43) | NA | NA | 5 |
| **267** | Ouellette-Kuntz, 2006 | Canada | Manitoba | North America | HI | 0.936 | 2002 | 10-14 | AD | DSM-IV | 77,392 | 77,392.00 | 186 | 186.00 | 24 (20.7–27.7) | NA | NA | 5 |
| **268** | Ouellette-Kuntz, 2006 | Canada | Prince Edward Island | North America | HI | 0.936 | 2002 | 1-4 | AD | DSM-IV | 5,972 | 5,972.00 | 9 | 9.00 | 15.1 (6.9–28.6) | NA | NA | 5 |
| **269** | Ouellette-Kuntz, 2006 | Canada | Prince Edward Island | North America | HI | 0.936 | 2002 | 5-9 | AD | DSM-IV | 8,776 | 8,776.00 | 36 | 36.00 | 41.0 (28.7–56.7) | NA | NA | 5 |
| **270** | Ouellette-Kuntz, 2006 | Canada | Prince Edward Island | North America | HI | 0.936 | 2002 | 10-14 | AD | DSM-IV | 9,967 | 9,967.00 | 42 | 42.00 | 42.1 (30.4–56.9) | NA | NA | 5 |
| **272** | Mattila, 2011 | Finland | North. Ostrobothnia | Europe | HI | 0.940 | 2000 | 8 | Mixed (DS&AD) | DSM-IV-TR | 4,422 | 4,422.00 | 37 | 37.00 | 83.7 (61–115) | 1.8 | 35.0 | 2 |
| **275** | Van Naarden Braun, 2015 | USA | Metropolitan Atlanta | North America | HI | 0.921 | 1996 | 8 | RRS | DSM-IV-TR | 36,749 | 36,749.00 | 156 | 156.00 | 42.5 (NA) | 3.9 | 58.5 | 5 |
| **276** | Van Naarden Braun, 2015 | USA | Metropolitan Atlanta | North America | HI | 0.921 | 2000 | 8 | RRS | DSM-IV-TR | 43,593 | 43,593.00 | 285 | 285.00 | 65.4 (NA) | 5.5 | 36.6 | 5 |
| **277** | Van Naarden Braun, 2015 | USA | Metropolitan Atlanta | North America | HI | 0.921 | 2002 | 8 | RRS | DSM-IV-TR | 43,201 | 43,201.00 | 337 | 337.00 | 78.0 (NA) | 4.9 | 46.2 | 5 |
| **278** | Van Naarden Braun, 2015 | USA | Metropolitan Atlanta | North America | HI | 0.921 | 2004 | 8 | RRS | DSM-IV-TR | 42,363 | 42,363.00 | 401 | 401.00 | 94.7 (NA) | 4.1 | 34.4 | 5 |
| **279** | Van Naarden Braun, 2015 | USA | Metropolitan Atlanta | North America | HI | 0.921 | 2006 | 8 | RRS | DSM-IV-TR | 43,241 | 43,241.00 | 474 | 474.00 | 109.6 (NA) | 5.2 | 34.6 | 5 |
| **280** | Van Naarden Braun, 2015 | USA | Metropolitan Atlanta | North America | HI | 0.921 | 2008 | 8 | RRS | DSM-IV-TR | 45,248 | 45,248.00 | 601 | 601.00 | 132.8 (NA) | 5.5 | 39.6 | 5 |
| **281** | Van Naarden Braun, 2015 | USA | Metropolitan Atlanta | North America | HI | 0.921 | 2010 | 8 | RRS | DSM-IV-TR | 48,529 | 48,529.00 | 752 | 752.00 | 155 (NA) | 4.7 | 35.7 | 5 |
| **282** | Chakrabarti, 2001 | UK | Stafford, Cannock, Wightwick | Europe | HI | 0.929 | 1998 | 2,5-6,5 | DS | DSM-IV | 15,500 | 15,500.00 | 97 | 97.00 | 62.6 (50.8–76.3) | 3.9 | 25.8 | 2 |
| **286** | Kielinen, 2000 | Finland | Oulu, Lapland | Europe | HI | 0.940 | 1996 | 5-7 | AD | ICD-10, DSM-IV | 27,572 | 27,572.00 | 63 | 63.00 | 22.8 (17.2–28.5) | NA | NA | 4 |
| **288** | Kielinen, 2000 | Finland | Oulu, Lapland | Europe | HI | 0.940 | 1996 | 15-18 | AD | ICD-10, DSM-IV | 39,216 | 39,216.00 | 30 | 30.00 | 7.6 (4.9–10.4) | NA | 46.7 | 4 |
| **290** | Shaw, 2020 | USA | 6 sites | North America | HI | 0.921 | 2016 | 4 | RRS | DSM-5 | 72,277 | 72,277.00 | 1,125 | 1,125.00 | 155.7 (147–165) | 3.7 | 53.0 | 5 |
| **291** | Kakooza-Mwesige, 2014 | Uganda | Kampala, Wakiso | Africa | LI | 0.525 | 2010 | 2-9 | DS | DSM-IV-TR | 1,169 | 1,169.00 | 8 | 14.03 | 120.0 (NA) | NA | NA | 5 |
| **297** | Fernell, 2010 | Sweden | Stockholm | Europe | HI | 0.947 | 2008 | 6 | AD | DSM-IV (TR), ICD-10 | 23,566 | 23,566.00 | 147 | 147.00 | 62.4 (52–72) | 5.1 | 34.5 | 3 |
| **301** | Surén, 2012 | Norway | Norway | Europe | HI | 0.961 | 2010 | 6-11 | Reg | ICD-10 | 362,738 | 362,738.00 | 1,415 | 1,415.00 | 39.0 (NA) | 4.3 | NA | 4 |
| **302** | Croen, 2007 | USA | North. California | North America | HI | 0.921 | 2005 | 5-10 | AD | ICD-9-CM | 132,844 | 132,844.00 | 593 | 593.00 | 44.6 (NA) | 5.4 | NA | 4 |
| **303** | Lai, 2013 | Taiwan | Taiwan | Asia | HI | NA | 2000 | 3-7 | Reg | DSM-IV (TR) | 4,936,300 | 4,936,300.00 | 1,836 | 1,836.00 | 3.7 (NA) | NA | NA | 4 |
| **304** | Lai, 2013 | Taiwan | Taiwan | Asia | HI | NA | 2001 | 3-7 | Reg | DSM-IV (TR) | 4,826,984 | 4,826,984.00 | 2,266 | 2,266.00 | 4.7 (NA) | NA | NA | 4 |
| **305** | Lai, 2013 | Taiwan | Taiwan | Asia | HI | NA | 2002 | 3-7 | Reg | DSM-IV (TR) | 4,744,217 | 4,744,217.00 | 2,780 | 2,780.00 | 5.9 (NA) | NA | NA | 4 |
| **306** | Lai, 2013 | Taiwan | Taiwan | Asia | HI | NA | 2003 | 3-7 | Reg | DSM-IV (TR) | 4,709,053 | 4,709,053.00 | 3,324 | 33,24l.00 | 7.1 (NA) | NA | NA | 4 |
| **307** | Lai, 2013 | Taiwan | Taiwan | Asia | HI | NA | 2004 | 3-7 | Reg | DSM-IV (TR) | 4,664,310 | 4,664,310.00 | 3,995 | 3,995.00 | 8.6 (NA) | NA | NA | 4 |
| **308** | Lai, 2013 | Taiwan | Taiwan | Asia | HI | NA | 2005 | 3-7 | Reg | DSM-IV (TR) | 4,601,833 | 4,601,833.00 | 4,684 | 4,684.00 | 10.2 (NA) | NA | NA | 4 |
| **309** | Lai, 2013 | Taiwan | Taiwan | Asia | HI | NA | 2006 | 3-7 | Reg | DSM-IV (TR) | 4,487,827 | 4,487,827.00 | 5,345 | 5,345.00 | 11.9 (NA) | NA | NA | 4 |
| **310** | Lai, 2013 | Taiwan | Taiwan | Asia | HI | NA | 2007 | 3-7 | Reg | DSM-IV (TR) | 4,395,283 | 4,395,283.00 | 6,119 | 6,119.00 | 13.9 (NA) | NA | NA | 4 |
| **311** | Lai, 2013 | Taiwan | Taiwan | Asia | HI | NA | 2008 | 3-7 | Reg | DSM-IV (TR) | 4,268,630 | 4,268,630.00 | 6,771 | 6,771.00 | 15.9 (NA) | NA | NA | 4 |
| **312** | Lai, 2013 | Taiwan | Taiwan | Asia | HI | NA | 2009 | 3-7 | Reg | DSM-IV (TR) | 4,157,940 | 4,157,940.00 | 7,479 | 7,479.00 | 18.0 (NA) | NA | NA | 4 |
| **313** | Lai, 2013 | Taiwan | Taiwan | Asia | HI | NA | 2010 | 3-7 | Reg | DSM-IV (TR) | 4,044,433 | 4,044,433.00 | 8,072 | 8,072.00 | 20.0 (NA) | NA | NA | 4 |
| **314** | Lai, 2013 | Taiwan | Taiwan | Asia | HI | NA | 2011 | 3-7 | Reg | DSM-IV (TR) | 3,917,643 | 3,917,643.00 | 8,671 | 8,67100 | 22.1 (NA) | NA | NA | 4 |
| **315** | Sun, 2014 | UK | Cambridgeshire | Europe | HI | 0.929 | 2003 | 5-10 | Mixed (DS&AD) | ICD-10 | 3,329 | 3,329.00 | 46 | 46.00 | 138.2 (NA) | 3.2 | NA | 5 |
| **316** | Samadi, 2015 | Iran | Mahabad | Asia | LMI | 0.774 | 2010 | 2-5 | DS | ADI-R, ADOS | 2,941 | 2,941.00 | 28 | 28.00 | 95.2 (66–137) | NA | NA | 2 |

*Est. ID, Estimation Identificator; HDI,* Human Development Index; *RoB, Risk of Bias; HI, High Income; UMI, Upper Middle Income; LMI, Lower Middle Income; LI, Low Income; Reg, Register; AD, Administrative Databases; DS, Direct Surveillance, RRS, Records-review Surveillance, HID, Health Insurance Databases;* *SEC, Special Education Criteria; NA, Not Available*

**Supplementary Table 2.** Data for Autism Disorder included in the meta-analysis

| **Est. ID** | **Author, Year** | **Country** | **Area** | **Geographical region** | **Income** | **HDI** | **Study time** | **Age, years** | **Design** | **Case criterion** | **Sample Size** | **Sample Size for MA** | **Number of Cases** | **Number of Cases for MA** | **Prevalence per 10000 (95% CI)** | **Gender ratio** | **IQ Score < 70 (%)** | **RoB** |
| --- | --- | --- | --- | --- | --- | --- | --- | --- | --- | --- | --- | --- | --- | --- | --- | --- | --- | --- |
| **4** | Parner, 2011 | Denmark | Denmark | Europe | HI | 0.948 | 2004 | 5-6 | Reg | ICD-10 | 131,842 | 122,274.90 | 258 | 258.00 | 21.0 (18,6- 23,9) | 5.3 | NA | 4 |
| **5** | Parner, 2011 | Denmark | Denmark | Europe | HI | 0.948 | 2004 | 7-8 | Reg | ICD-10 | 134,505 | 128,947.40 | 245 | 245.00 | 19.0 (16,7-21,5) | 4.2 | NA | 4 |
| **6** | Parner, 2011 | Denmark | Denmark | Europe | HI | 0.948 | 2004 | 9-10 | Reg | ICD-10 | 138,469 | 135,384.60 | 264 | 264.00 | 20.0 (17,3-22,0) | 4.3 | NA | 4 |
| **10** | Parner, 2011 | Australia | West. Australia | Oceania | HI | 0.951 | 2004 | 5-6 | Reg | DSM-IV (TR) | 50,803 | 49,714.30 | 174 | 174.00 | 35.0 (30.2-40.7) | 4.3 | NA | 4 |
| **11** | Parner, 2011 | Australia | West. Australia | Oceania | HI | 0.951 | 2004 | 7-8 | Reg | DSM-IV (TR) | 50,125 | 48,294.00 | 184 | 184.00 | 38.1 (32.9-44.1) | 4.3 | NA | 4 |
| **12** | Parner, 2011 | Australia | West. Australia | Oceania | HI | 0.951 | 2004 | 9-10 | Reg | DSM-IV (TR) | 50,120 | 49,529.80 | 158 | 158.00 | 31.9 (27.3-37.3) | 7.3 | NA | 4 |
| **24** | Ellefsen, 2007 | Denmark | Faroe Island | Europe | HI | 0.948 | 2002 | 7-16 | Mixed (DS&AD) | ICD-10 | 7,689 | 7,689.00 | 12 | 12.00 | 16.0 (7-25) | 3.0 | 91.7 | 2 |
| **43** | Zahorodny, 2014 | USA | New Jersey | North America | HI | 0.921 | 2006 | 8 | RRS | DSM-IV-TR | 30,570 | 30,570.00 | 413 | 413.00 | 135.1 (NA) | NA | NA | 5 |
| **47** | Honda, 2005 | Japan | Yokohama | Asia | HI | 0.925 | 1994 | 5 | AD | ICD-10 | 8,537 | 8,537.00 | 18 | 18.00 | 21.1 (NA) | NA | NA | 3 |
| **48** | Honda, 2005 | Japan | Yokohama | Asia | HI | 0.925 | 1995 | 5 | AD | ICD-10 | 7,857 | 7,857.00 | 30 | 30.00 | 38.2 (NA) | NA | NA | 3 |
| **49** | Honda, 2005 | Japan | Yokohama | Asia | HI | 0.925 | 1996 | 5 | AD | ICD-10 | 8,147 | 8,147.00 | 41 | 41.00 | 50.3 (NA) | NA | NA | 3 |
| **50** | Honda, 2005 | Japan | Yokohama | Asia | HI | 0.925 | 1997 | 5 | AD | ICD-10 | 8,250 | 8,250.00 | 34 | 34.00 | 41.2 (NA) | NA | NA | 3 |
| **59** | Montiel-Nava, 2008 | Venezuela | Maracaibo County | South America | NA | 0.691 | 2005 | 3-5 | AD | DSM-IV-TR | 122,877 | 122,877.00 | 150 | 150.00 | 12.0 (10-14) | 4.2 | NA | 3 |
| **61** | Montiel-Nava, 2008 | Venezuela | Maracaibo County | South America | NA | 0.691 | 2005 | 6-9 | AD | DSM-IV-TR | 132,028 | 132,028.00 | 137 | 137.00 | 10.4 (7-1,3) | 4.1 | NA | 3 |
| **65** | Tebruegge, 2004 | UK | Kent | Europe | HI | 0.929 | 2000 | 8-9 | AD | ICD-10 | 2,536 | 2,536.00 | 6 | 6.00 | 23.7 (NA) | NA | NA | 3 |

*Est. ID, Estimation Identificator; HDI,* Human Development Index; *RoB, Risk of Bias; HI, High Income; UMI, Upper Middle Income; LMI, Lower Middle Income; LI, Low Income; Reg, Register; AD, Administrative Databases; DS, Direct Surveillance, RRS, Records-review Surveillance, HID, Health Insurance Databases;* *SEC, Special Education Criteria; NA, Not Available*

**Supplementary Table 3.** Data for Asperger Syndrome included in the meta-analysis

| **Est. ID** | **Author, Year** | **Country** | **Area** | **Geographical region** | **Income** | **HDI** | **Study time** | **Age, years** | **Design** | **Case criterion** | **Sample Size** | **Sample Size for MA** | **Number of Cases** | **Number of Cases for MA** | **Prevalence per 10000 (95% CI)** | **Gender ratio** | **IQ Score < 70 (%)** | **RoB** |
| --- | --- | --- | --- | --- | --- | --- | --- | --- | --- | --- | --- | --- | --- | --- | --- | --- | --- | --- |
| **25** | Ellefsen, 2007 | Denmark | Faroe Island | Europe | HI | 0.948 | 2002 | 7-16 | Mixed (DS& AD) | Gillberg criteria | 7,689 | 7,689.00 | 20 | 20.00 | 26.0 (14-38) | 6.0 | 10.0 | 2 |
| **66** | Tebruegge, 2004 | UK | Kent | Europe | HI | 0.929 | 2000 | 8-9 | AD | ICD-10, Gillberg criteria | 2,536 | 2,536.00 | 3 | 3.00 | 11.8 (NA) | NA | NA | 3 |
| **69** | van Bakel, 2015 | France | Haute-Garonne, Isere, Savoy, Upper-Savoy counties | Europe | HI | 0.903 | 2004 | 7 | Reg | ICD-10 | 42,360 | 42,360.00 | 5 | 5.00 | 1.2 (NA) | NA | NA | 4 |
| **72** | van Bakel, 2015 | France | Haute-Garonne, Isere, Savoy, Upper-Savoy counties | Europe | HI | 0.903 | 2005 | 7 | Reg | ICD-10 | 42,724 | 42,724.00 | 5 | 5.00 | 1.2 (NA) | NA | NA | 4 |
| **75** | van Bakel, 2015 | France | Haute-Garonne, Isere, Savoy, Upper-Savoy counties | Europe | HI | 0.903 | 2006 | 7 | Reg | ICD-10 | 43,977 | 43,977.00 | 10 | 10.00 | 2.3 (NA) | NA | NA | 4 |
| **78** | van Bakel, 2015 | France | Haute-Garonne, Isere, Savoy, Upper-Savoy counties | Europe | HI | 0.903 | 2007 | 7 | Reg | ICD-10 | 44,275 | 44,275.00 | 8 | 8.00 | 1.8 (NA) | NA | NA | 4 |
| **81** | van Bakel, 2015 | France | Haute-Garonne, Isere, Savoy, Upper-Savoy counties | Europe | HI | 0.903 | 2008 | 7 | Reg | ICD-10 | 44,645 | 44,645.00 | 9 | 9.00 | 2.0 (NA) | NA | NA | 4 |
| **84** | van Bakel, 2015 | France | Haute-Garonne, Isere, Savoy, Upper-Savoy counties | Europe | HI | 0.903 | 2009 | 7 | Reg | ICD-10 | 44,742 | 44,742.00 | 10 | 10.00 | 2.2 (NA) | NA | NA | 4 |
| **87** | van Bakel, 2015 | France | Haute-Garonne, Isere, Savoy, Upper-Savoy counties | Europe | HI | 0.903 | 2010 | 7 | Reg | ICD-10 | 45,028 | 45,028.00 | 6 | 6.00 | 1.3 (NA) | NA | NA | 4 |

*Est. ID, Estimation Identificator; HDI,* Human Development Index; *RoB, Risk of Bias; HI, High Income; UMI, Upper Middle Income; LMI, Lower Middle Income; LI, Low Income; Reg, Register; AD, Administrative Databases; DS, Direct Surveillance, RRS, Records-review Surveillance, HID, Health Insurance Databases;* *SEC, Special Education Criteria; NA, Not Available*

**Supplementary Table 4.** Data for Atypical Autism included in the meta-analysis

| **Est. ID** | **Author, Year** | **Country** | **Area** | **Geographical region** | **Income** | **HDI** | **Study time** | **Age, yrs** | **Design** | **Case criterion** | **Sample Size** | **Sample Size for MA** | **Number of Cases** | **Number of Cases for MA** | **Prevalence per 10000 (95% CI)** | **Gender ratio** | **IQ Score < 70 (%)** | **RoB** |
| --- | --- | --- | --- | --- | --- | --- | --- | --- | --- | --- | --- | --- | --- | --- | --- | --- | --- | --- |
| **26** | Ellefsen, 2007 | Denmark | Faroe Island | Europe | HI | 0.948 | 2002 | 7-16 | Mixed (DS&AD) | ICD-10 | 7,689 | 7,689.00 | 9 | 9.00 | 12.0 (4-20) | NA | 0 | 2 |
| **91** | Williams, 2008 | UK | Avon | Europe | HI | 0.929 | 2003 | 11 | AD | ICD-10, SEC | 14,062 | 13,898.00 | 15 | 15.00 | 10,8 (5.3-16.3) | 14.0 | 26.7 | 6 |
| **95** | Kočovská, 2012 | Denmark | Faroe Island | Europe | HI | 0.948 | 2009 | 15-24 | Mixed (DS&RRS) | Billstedt et al., 2005 criteria | 7,128 | 7,128.00 | 15 | 15.00 | 21.0 (12.0-35.0) | 6.5 | 20.0 | 2 |
| **99** | Lingam, 2003 | UK | London | Europe | HI | 0.929 | 2000 | 5-14 | Reg | ICD-10 | 186,206 | 18,620.,00 | 195 | 195.00 | 10.5 (NA) | 4.3 | NA | 4 |
| **193** | Magnússon, 2001 | Iceland | Iceland | Europe | HI | 0.959 | 1998 | 5-14 | Reg | ICD-9, ICD-10 | 43,153 | 43,153.00 | 20 | 20.00 | 4.6 (NA) | 5.7 | 65.0 | 2 |
| **195** | Magnússon, 2001 | Iceland | Iceland | Europe | HI | 0.959 | 1998 | 15-24 | Reg | ICD-9/10 | 42,403 | 42,403.00 | 2 | 2.00 | 0.5 (NA) | NA | NA | 3 |
| **220** | Isaksen, 2012 | Norway | Oppland, Hedmark | Europe | HI | 0.961 | 2008 | 6-12 | Mixed (Reg&RRS) | ICD-10 | 31,015 | 31,015.00 | 16 | 16.00 | 5.2 (3-8) | 4.3 | NA | 2 |
| **300** | Fernell, 2010 | Sweden | Stockholm | Europe | HI | 0.947 | 2008 | 6 | AD | DSM-IV (TR), ICD-10 | 23,566 | 23,566.00 | 53 | 53.00 | 22.5 (16-28) | 3.8 | 15.1 | 2 |

*Est. ID, Estimation Identificator; HDI,* Human Development Index; *RoB, Risk of Bias; HI, High Income; UMI, Upper Middle Income; LMI, Lower Middle Income; LI, Low Income; Reg, Register; AD, Administrative Databases; DS, Direct Surveillance, RRS, Records-review Surveillance, HID, Health Insurance Databases;* *SEC, Special Education Criteria; NA, Not Available*

**Supplementary Table 5.** Data for Pervasive Developmental Disorder-Not Otherwise Specified included in the meta-analysis

| **Est. ID** | **Author, Year** | **Country** | **Area** | **Geographical region** | **Income** | **HDI** | **Study time** | **Age, yrs** | **Design** | **Case criterion** | **Sample Size** | **Sample Size for MA** | **Number of Cases** | **Number of Cases for MA** | **Prevalence per 10000 (95% CI)** | **Gender ratio** | **IQ Score < 70 (%)** | **RoB** |
| --- | --- | --- | --- | --- | --- | --- | --- | --- | --- | --- | --- | --- | --- | --- | --- | --- | --- | --- |
| **177** | Aguilera, 2007 | Spain | Seville | Europe | HI | 0.905 | 2002 | 3-21 | AD | DSM-IV | 127,350 | 127,350.00 | 55.0 | 55.00 | 4.3 (NA) | NA | NA | 4 |
| **183** | Gillberg, 2006 | Sweden | Gothenburg | Europe | HI | 0.947 | 2001 | 7-12 | Mixed (Reg&RRS) | DSM-IV, clinical interpretation | 32,568 | 32,568.00 | 124.1 | 124.10 | 38.1 (NA) | 3.4 | NA | 4 |
| **187** | Gillberg, 2006 | Sweden | Gothenburg | Europe | HI | 0.947 | 2001 | 13-18 | Mixed (Reg&RRS) | DSM-IV, clinical interpretation | 28,507 | 28,507.00 | 81.0 | 81.00 | 28.4 (NA) | 3.8 | NA | 4 |
| **191** | Gillberg, 2006 | Sweden | Gothenburg | Europe | HI | 0.947 | 2001 | 19-24 | Mixed (Reg&RRS) | DSM-IV, clinical interpretation | 41,410 | 41,410.00 | 36.0 | 36.00 | 8.7 (NA) | 2.7 | NA | 4 |
| **199** | Fombonne, 2006 | Canada | Montreal | North America | HI | 0.936 | 2003 | 5-17 | AD | DSM-IV | 27,749 | 27,749.00 | 91.0 | 91.00 | 32.8 (26.4-40.2) | 6.6 | NA | 5 |
| **204** | Chakrabarti, 2005 | UK | Stafford, Cannock | Europe | HI | 0.929 | 2002 | 4-6 | DS | DSM-IV | 10,903 | 10,903.00 | 27.0 | 27.00 | 24.8 (16.3-36.0) | 5.8 | 12.0 | 1 |
| **216** | Saemundsen, 2013 | Iceland | Iceland | Europe | HI | 0.959 | 2009 | 11-15 | Reg | ICD-10 | 22,229 | 22,229.00 | 144.0 | 144.00 | 64.8 (55.1-76.2) | 3.2 | 46.5 | 0 |
| **264** | Nygren, 2012 | Sweden | Gothenburg | Europe | HI | 0.947 | 2010 | 2 | AD | DSM-IV-TR | 5,007 | 5,007.00 | 14.0 | 14.00 | 28.0 (NA) | 2.5 | NA | 1 |
| **285** | Chakrabarti, 2001 | UK | Stafford, Cannock, Wightwick | Europe | HI | 0.929 | 1998 | 2.5-6.5 | DS | DSM-IV | 15,500 | 15,500.00 | 56.0 | 56.00 | 36.1 (27.3-46.9) | NA | 7.6 | 1 |

*Est. ID, Estimation IDentificator; HDI,* Human Development Index; *RoB, Risk of Bias; HI, High Income; UMI, Upper Middle Income; LMI, Lower Middle Income; LI, Low Income; Reg, Register; AD, Administrative Databases; DS, Direct Surveillance, RRS, Records-review Surveillance, HID, Health Insurance Databases;* *NA, Not Available*

**Supplementary Table 6.** Summary of studies included in the meta-analysis

| **Group** | **ASD** | **AD** | **AS** | **AA** | **PDD-NOS** |
| --- | --- | --- | --- | --- | --- |
| Study years | 1996 - 2019 | 1994 - 2010 | 1998 - 2010 | 1998 - 2009 | 1998-2010 |
| Number of studies | 79 | 30 | 15 | 7 | 7 |
| Number of estimates | 220 | 57 | 23 | 8 | 9 |
| Overall sample size | 116,039,044.6 | 2,534,516 | 890,591 | 355,222 | 311,223 |
| Range of sample sizes | 1,169 - 6,900,000 | 1,294 - 186,206 | 2,536 - 186,206 | 7,128 - 186,206 | 5,007 - 127,350 |
| Overall number of cases | 271,517.7 | 4,819.9 | 553.1 | 325 | 628.1 |
| Range of number of cases | [0 - 21,286] | [0 - 413] | [3 - 94] | [2 - 195] | [14 - 144] |
| **Age, years** |  |  |  |  |  |
| Mean ± SD  Median | 7.8**±**4  8.0 | 9.6**±**4.5   8.3 | 9.7**±**4.4   8.3 | 12.4**±**5.7  10.3 | 10.4**±**6.1   11.0 |
| **Male to female ratio:** |  |  |  |  |  |
| Mean±SD   Median | 4.3**±**1.7   4.1 | 4.2**±**1.8   4.2 | 10.1**±**8.3   7.6 | 6.6**±**4.3   4.3 | 4.0**±**1.6  3.4 |
| **IQ<70, %:** |  |  |  |  |  |
| Mean±SD  Median | 32.3**±**12.6   31.2 | 56.9**±**22.5  60.0 | 2.8**±**4.0  0 | 20.6**±**5.8  20.0 | 20.0**±**21.3   12.0 |
| **Geographic regions** |  |  |  |  |  |
| **Africa:**                                  studies  estimates  countries | 1  1  1 | NA  NA  NA | NA  NA  NA | NA  NA  NA | NA  NA  NA |
| **Asia:**  studies  estimates  countries | 18  87 11 | 4  11  4 | NA  NA  NA | NA  NA  NA | NA  NA  NA |
| **Europe:**  studies  estimates  countries | 33  65  12 | 20  36  9 | 14  22  9 | 7  8  5 | 6  8  4 |
| **North America:**  studies  estimates  countries | 25  62  3 | 4  5  2 | 1  1  1 | NA  NA  NA | 1  1  1 |
| **Oceania:**  studies  estimates  countries | 1  3  1 | 1  3  1 | NA  NA  NA | NA  NA  NA | NA  NA  NA |
| **South America:**  studies  estimates  countries | 1  2  1 | 1  2  1 | NA  NA  NA | NA  NA  NA | NA  NA  NA |
| **Study design** |  |  |  |  |  |
| **Administrative database:**  studies  estimates | 19   47 | 9  14 | 5  5 | 2  2 | 3  3 |
| **Direct surveillance:**  studies  estimates | 15   27 | 7  12 | 2  2 | NA  NA | 2  2 |
| **Health insurance database:**  studies  estimates | 4  14 | NA  NA | NA  NA | NA  NA | NA  NA |
| **Records review surveillance:**  studies  estimates | 16  23 | 2  2 | NA  NA | NA  NA | NA  NA |
| **Register database:**  Studies:  estimates | 13   88 | 6   20 | 3  9 | 2  3 | 1  1 |
| **Mixed (direct surveillance, administrative databases):**  studies  estimates | 6  12 | 2  2 | 2  2 | 1  1 | NA  NA |
| **Mixed (direct surveillance, records review surveillance):**  studies  estimates | 4  5 | 2  3 | 1  1 | 1  1 | NA  NA |
| **Mixed (register, records review surveillance):**  studies  estimates | 2  4 | 2  4 | 2  4 | 1  1 | 1  3 |

SD, Standard deviation

**Supplementary Table 7.** Model fit statistics for two- and three-level models

|  | | **AIC** | **BIC** | **LRT** |
| --- | --- | --- | --- | --- |
| **ASD** | | | |  |
| Three-level model | | -1019.9369 | -1009.7697 |  |
| Two-level model | | -828.5692 | -821.7910 | 193.3677*** |
| **AD** | | | | |
| Three-level model | | -304.6278 | -293.1386 |  |
| Two-level model | | -283.8632 | -274.4938 | 22.7645*** |
| **AS** | | | |  |
| Three-level model | | -133.1990 | -129.9258 |  |
| Two-level model | | -113.4889 | -111.3068 | 21.7101*** |
| **PDD-NOS&AA** | | | |  |
| Three-level model | | -76.4978 | -74.1800 |  |
| Two-level model | -77.6286 | -76.0834 | 0.8692 |  |

AIC, Akaike Information Criterion; BIC, Bayesian Information Criterion; LRT, Likelihood Ratio Test; ASD, Autism spectrum Disorder (s); AD, Autistic Disorder, AS, Asperger Disorder; PDD-NOS&AA, combined group of Pervasive Developmental Disorder-Not Otherwise Specified and Atypical Autism; ***, p-value < .001

**Supplementary Table 8.** Distribution of variance across levels

| **Diagnostic group** | **I^2^_Level1_, %** | **I^2^_Level2_, %** | **I^2^_Level3_, %** | **I^2^_Total_, %** |
| --- | --- | --- | --- | --- |
| ASD | 0.04 | 22.88 | 77.08 | 99.96 |
| AD | 1.30 | 14.19 | 84.51 | 98.70 |
| AS | 2.57 | 2.34 | 95.09 | 97.43 |
| AA&PDD-NOS | 1.97 | 53.23 | 44.80 | 98.03 |

ASD, Autism spectrum Disorder (s); AD, Autistic Disorder, AS, Asperger Disorder; PDD-NOS&AA, combined group of Pervasive Developmental Disorder-Not Otherwise Specified and Atypical Autism

**Supplementary Table 9.** Results for moderator analysis for ASD

| **Moderator variable** | **Studies, №** | **DP, №** | **Intercept (95% CI) / Mean Z (95% CI)** | **Prevalence per 10,000 (95% CI)** | **β (95% CI)** | **Test of Moderators** | **Test for Residual Heterogeneity** | **I^2^_Level2_, %** | **I^2^_Level3_, %** | ***Total variance,  I*^2^, %** |
| --- | --- | --- | --- | --- | --- | --- | --- | --- | --- | --- |
| **Income** |  |  |  |  |  | *F*(3,214) = 199.599*** | *QE*(214) = 251988.308*** | 24.71 | 75.24 | 99.95 |
| High income | 67 | 190 | 0.090 (0.082, 0.097)*** | 79 (67–93) |  |  |  |  |  |  |
| Lower middle income | 6 | 9 | 0.057 (0.029, 0.085 )*** | 32 (8–71) | -0.033 (-0.061–-0.004)* |  |  |  |  |  |
| Upper middle income | 5 | 19 | 0.064 (0.038, 0.091)*** | 41 (14–83) | -0.025 (0.078–0.003) |  |  |  |  |  |
| **Region** |  |  |  |  |  | *F*(2,211) = 8.579*** | QE(211) = 108119.076*** | 28.46 | 71.49 | 99.95 |
| Asia | 18 | 87 | 0.064 (0.051, 0.077)*** | 41 (26–59) |  |  |  |  |  |  |
| Europe | 32 | 65 | 0.086 (0.076, 0.096)*** | 73 (57–91) | 0.022 (0.005–0.038)* |  |  |  |  |  |
| North America | 26 | 62 | 0.101 (0.089, 0.112)*** | 101 (79–125) | 0.035 (0.037–0.019)*** |  |  |  |  |  |
| **Country** |  |  |  |  |  | *F*(9, 173) =6.650*** | *QE*( 173) = 48960.017*** | 39.33 | 60.57 | 99.90 |
| Canada | 6 | 28 | 0.085 (0.068, 0.101***) | 71 (46–101) |  |  |  |  |  |  |
| China | 4 | 18 | 0.063 (0.043, 0.083***) | 39 (18–68) | -0.022 (-0.048–-0.004 ) |  |  |  |  |  |
| Denmark | 5 | 11 | 0.086 (0.070, 0.102)*** | 73 (49–104) | 0.001 (-0.022–0.024) |  |  |  |  |  |
| Finland | 3 | 6 | 0.065 (0.046, 0.084)*** | 42 (21–70) | -0.020 (-0.045–0.005) |  |  |  |  |  |
| France | 2 | 9 | 0.057 (0.036, 0.080)*** | 32 (12–61) | -0.028(-0.0545–-0.001)* |  |  |  |  |  |
| Spain | 3 | 5 | 0.073 (0.048, 0.097)**** | 52 (22–93) | -0.012 (-0.042–0.019)) |  |  |  |  |  |
| Sweden | 5 | 13 | 0.095 (0.077, 0.114)*** | 90 (59–127) | 0.010(-0.014–0.035) |  |  |  |  |  |
| Taiwan | 3 | 50 | 0.034 (0.014, 0.055)** | 11 (2–29) | -0.051 (-0.078–-0.025 )*** |  |  |  |  |  |
| UK | 10 | 10 | 0.082 (0.068, 0.097)*** | 67 (45–92) | -0.003(-0.024–0.019) |  |  |  |  |  |
| USA | 19 | 33 | 0.106 (0.096, 0.116)*** | 112 (92–133) | 0.021 (0.002–0.040)* |  |  |  |  |  |
| **Study Time Ranges** |  |  |  |  |  | *F*(4, 215) = 8.935*** | *QE*(215) = 131985.522*** | 22.93 | 77.02 | 99.95 |
| 1994-1999 | 6 | 11 | 0.050 (0.036, 0.065)*** | 25 (12, 42) |  |  |  |  |  |  |
| 2000-2004 | 23 | 48 | 0.070 (0.062, 0.079)*** | 49 (37, 62) | 0.020 (0.006, 0.034)** |  |  |  |  |  |
| 2005-2009 | 26 | 80 | 0.081 (0.073, 0.089)*** | 65 (53, 79) | 0.031 (0.016, 0.046)*** |  |  |  |  |  |
| 2010-2014 | 26 | 53 | 0.091 (0.082, 0.100)*** | 83 (67, 99) | 0.041 (0.025, 0.057)*** |  |  |  |  |  |
| 2015-2019 | 14 | 28 | 0.100 (0.086, 0.114)*** | 99 (73, 128) | 0.049 (0.030, 0.069)*** |  |  |  |  |  |
| **Age groups** |  |  |  |  |  | *F*(2, 159) = 6.109** | *QE*(159) = 109956.860*** | 24.42 | 75.47 | 99.89 |
| >13 | 44 | 98 | 0.076 (0.064,   0.088)*** | 57 (40, 76) |  |  |  |  |  |  |
| 0-5 | 23 | 46 | 0.078 (0.0678,  0.088)*** | 60 (45, 76) | -0.002 ( -0.010,  0.014)* |  |  |  |  |  |
| 6-12 | 8 | 21 | 0.091 (0.082,  0.099)*** | 82 (67, 98) | 0.015(-0.004,  0.026)** |  |  |  |  |  |
| **Study Design** |  |  |  |  |  | *F*(5, 214) = 4.083** | *QE*(214) =167848.718*** | 22.58 | 77.37 | 99.96 |
| Administrative Databases | 19 | 47 | 0.070(0.056, 0.083 )*** | 48 (31, 68) |  |  |  |  |  |  |
| Direct Surveillance | 15 | 27 | 0.086 (0.071,  0.102)*** | 75 (51, 103) | 0.016 (-0.004, 0.037) |  |  |  |  |  |
| Health Insurance Databases | 4 | 14 | 0.060 (0.032,  0.088)*** | 35 (10, 77) | -0.010  (-0.04, 0.022) |  |  |  |  |  |
| Mixed | 12 | 21 | 0.090 (0.073,  0.107)*** | 80 (52, 114) | 0.020 (-0.001, 0.042) |  |  |  |  |  |
| Records-review surveillance | 16 | 23 | 0.111 (0.096,  0.126)*** | 122 (91, 156) | 0.041(0.021, 0.061)*** |  |  |  |  |  |
| Register | 13 | 88 | 0.082 (0.067,  0.097)*** | 66 (44, 93) | 0.012 (-0.008, 0.032) |  |  |  |  |  |
| **Risk of Bias** |  |  |  |  |  | *F*(2, 217) = 0.345 | QE(217) = 202284.969* | 22.67 | 77.28 | 99.96 |
| High | 2 | 8 | 0.104 (0.060, 0.147)*** | 106 (35, 215) |  |  |  |  |  |  |
| Low | 25 | 53 | 0.086 (0.073, 0.099)*** | 73 (53, 97) | -0.018 (-0.063,  0.028) |  |  |  |  |  |
| Moderate | 52 | 159 | 0.085 (0.076, 0.093)*** | 71 (57, 87) | -0.019 (-0.064, 0.026) |  |  |  |  |  |

*, p-value < .05; **, p-value < .01; ***, p-value < .001


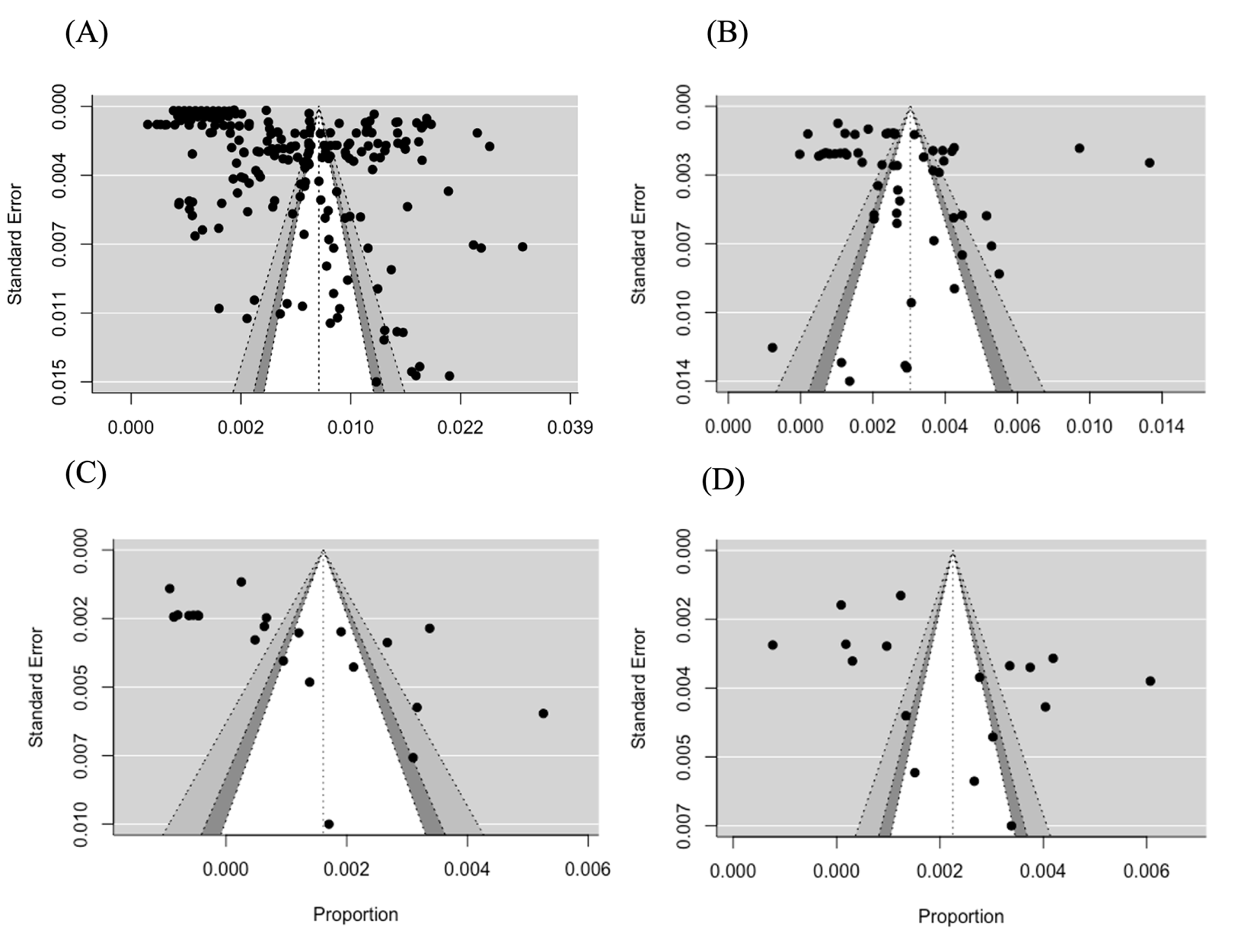


**Supplementary Figure 1.** Funnel Plots for Autism Spectrum Disorder (A), Autism Disorder (B), Asperger Syndrome (C) and Atypical Autism and Pervasive Developmental Disorder-Not Otherwise Specified (D)
